# Bilineage embryo-like structure from EPS cells can produce live mice with tetraploid trophectoderm

**DOI:** 10.1093/procel/pwac029

**Published:** 2022-07-15

**Authors:** Kuisheng Liu, Xiaocui Xu, Dandan Bai, Yanhe Li, Yalin Zhang, Yanping Jia, Mingyue Guo, Xiaoxiao Han, Yingdong Liu, Yifan Sheng, Xiaochen Kou, Yanhong Zhao, Jiqing Yin, Sheng Liu, Jiayu Chen, Hong Wang, Yixuan Wang, Wenqiang Liu, Shaorong Gao

**Affiliations:** Shanghai Key Laboratory of Maternal Fetal Medicine, Clinical and Translational Research Center of Shanghai First Maternity and Infant Hospital, Frontier Science Center for Stem Cell Research, School of Life Sciences and Technology, Tongji University, Shanghai 200092, China; Shanghai Key Laboratory of Maternal Fetal Medicine, Clinical and Translational Research Center of Shanghai First Maternity and Infant Hospital, Frontier Science Center for Stem Cell Research, School of Life Sciences and Technology, Tongji University, Shanghai 200092, China; Shanghai Key Laboratory of Maternal Fetal Medicine, Clinical and Translational Research Center of Shanghai First Maternity and Infant Hospital, Frontier Science Center for Stem Cell Research, School of Life Sciences and Technology, Tongji University, Shanghai 200092, China; Shanghai Key Laboratory of Maternal Fetal Medicine, Clinical and Translational Research Center of Shanghai First Maternity and Infant Hospital, Frontier Science Center for Stem Cell Research, School of Life Sciences and Technology, Tongji University, Shanghai 200092, China; Shanghai Key Laboratory of Maternal Fetal Medicine, Clinical and Translational Research Center of Shanghai First Maternity and Infant Hospital, Frontier Science Center for Stem Cell Research, School of Life Sciences and Technology, Tongji University, Shanghai 200092, China; Shanghai Key Laboratory of Maternal Fetal Medicine, Clinical and Translational Research Center of Shanghai First Maternity and Infant Hospital, Frontier Science Center for Stem Cell Research, School of Life Sciences and Technology, Tongji University, Shanghai 200092, China; Shanghai Key Laboratory of Maternal Fetal Medicine, Clinical and Translational Research Center of Shanghai First Maternity and Infant Hospital, Frontier Science Center for Stem Cell Research, School of Life Sciences and Technology, Tongji University, Shanghai 200092, China; Shanghai Key Laboratory of Maternal Fetal Medicine, Clinical and Translational Research Center of Shanghai First Maternity and Infant Hospital, Frontier Science Center for Stem Cell Research, School of Life Sciences and Technology, Tongji University, Shanghai 200092, China; Shanghai Key Laboratory of Maternal Fetal Medicine, Clinical and Translational Research Center of Shanghai First Maternity and Infant Hospital, Frontier Science Center for Stem Cell Research, School of Life Sciences and Technology, Tongji University, Shanghai 200092, China; Shanghai Key Laboratory of Maternal Fetal Medicine, Clinical and Translational Research Center of Shanghai First Maternity and Infant Hospital, Frontier Science Center for Stem Cell Research, School of Life Sciences and Technology, Tongji University, Shanghai 200092, China; Shanghai Key Laboratory of Maternal Fetal Medicine, Clinical and Translational Research Center of Shanghai First Maternity and Infant Hospital, Frontier Science Center for Stem Cell Research, School of Life Sciences and Technology, Tongji University, Shanghai 200092, China; Shanghai Key Laboratory of Maternal Fetal Medicine, Clinical and Translational Research Center of Shanghai First Maternity and Infant Hospital, Frontier Science Center for Stem Cell Research, School of Life Sciences and Technology, Tongji University, Shanghai 200092, China; Shanghai Key Laboratory of Maternal Fetal Medicine, Clinical and Translational Research Center of Shanghai First Maternity and Infant Hospital, Frontier Science Center for Stem Cell Research, School of Life Sciences and Technology, Tongji University, Shanghai 200092, China; Shanghai Key Laboratory of Maternal Fetal Medicine, Clinical and Translational Research Center of Shanghai First Maternity and Infant Hospital, Frontier Science Center for Stem Cell Research, School of Life Sciences and Technology, Tongji University, Shanghai 200092, China; Shanghai Key Laboratory of Maternal Fetal Medicine, Clinical and Translational Research Center of Shanghai First Maternity and Infant Hospital, Frontier Science Center for Stem Cell Research, School of Life Sciences and Technology, Tongji University, Shanghai 200092, China; Shanghai Key Laboratory of Maternal Fetal Medicine, Clinical and Translational Research Center of Shanghai First Maternity and Infant Hospital, Frontier Science Center for Stem Cell Research, School of Life Sciences and Technology, Tongji University, Shanghai 200092, China; Shanghai Key Laboratory of Maternal Fetal Medicine, Clinical and Translational Research Center of Shanghai First Maternity and Infant Hospital, Frontier Science Center for Stem Cell Research, School of Life Sciences and Technology, Tongji University, Shanghai 200092, China; Translational Medical Center for Stem Cell Therapy and Institute for Regenerative Medicine, Shanghai East Hospital, School of Life Sciences and Technology, Tongji University, Shanghai 200092, China; Shanghai Key Laboratory of Maternal Fetal Medicine, Clinical and Translational Research Center of Shanghai First Maternity and Infant Hospital, Frontier Science Center for Stem Cell Research, School of Life Sciences and Technology, Tongji University, Shanghai 200092, China; Shanghai Institute of Maternal-Fetal Medicine and Gynecologic Oncology, Shanghai First Maternity and Infant Hospital, Tongji University, Shanghai 200092, China; Shanghai Key Laboratory of Maternal Fetal Medicine, Clinical and Translational Research Center of Shanghai First Maternity and Infant Hospital, Frontier Science Center for Stem Cell Research, School of Life Sciences and Technology, Tongji University, Shanghai 200092, China; Translational Medical Center for Stem Cell Therapy and Institute for Regenerative Medicine, Shanghai East Hospital, School of Life Sciences and Technology, Tongji University, Shanghai 200092, China

**Keywords:** EPS cells, blastoid, primitive endoderm (PrE), trophectoderm (TE), *Gata6*

## Abstract

Self-organized blastoids from extended pluripotent stem (EPS) cells possess enormous potential for investigating postimplantation embryo development and related diseases. However, the limited ability of postimplantation development of EPS-blastoids hinders its further application. In this study, single-cell transcriptomic analysis indicated that the “trophectoderm (TE)-like structure” of EPS-blastoids was primarily composed of primitive endoderm (PrE)-related cells instead of TE-related cells. We further identified PrE-like cells in EPS cell culture that contribute to the blastoid formation with TE-like structure. Inhibition of PrE cell differentiation by inhibiting MEK signaling or knockout of *Gata6* in EPS cells markedly suppressed EPS-blastoid formation. Furthermore, we demonstrated that blastocyst-like structures reconstituted by combining the EPS-derived bilineage embryo-like structure (BLES) with either tetraploid embryos or tetraploid TE cells could implant normally and develop into live fetuses. In summary, our study reveals that TE improvement is critical for constructing a functional embryo using stem cells *in vitro*.

## Introduction

Totipotency is a transient state emerging only in the early stages of mammalian embryo development. Both the fertilized egg and the 2-cell blastomere of the mouse can independently give rise to a whole embryo including extraembryonic tissue. In contrast, a single blastomere at the 4- to 8-cell stage cannot support the development of the entire embryo, although blastomeres can contribute to the embryo and extraembryonic tissue in chimeras. Therefore, only the fertilized egg and 2-cell blastomere have totipotency (Tarkowski, 1959; Tarkowski and Wroblewska, 1967; Rossant, 1976; Papaioannou et al., 1989). In mice, due to the IN-OUT polarity of the embryo, starting from embryonic Day 2.5 (E2.5), the outer layer of cells gradually differentiate into trophectoderm (TE), which envelops the inner cell mass (ICM). The next cell fate determination occurs in the ICM of E3.5 embryo, which begins to give rise to the epiblast (EPI) and the primitive endoderm (PrE). After implantation, the EPI develops into the fetus. PrE differentiates into parietal endoderm (PE) and visceral endoderm (VE) and eventually becomes the main component of the parietal yolk sac and visceral yolk sac. TE first develops into trophoblast giant cells (TGCs), extraembryonic ectoderm (ExE), and ectoplacental cone (EPC) and finally into the placenta. Compared with the embryonic tissue developed by the EPI, PrE and TE both contributed to extraembryonic tissues (Cockburn and Rossant, 2010; Leung et al., 2016; Rossant and Tam, 2017; Baker and Pera, 2018).

Multiple cell lines that maintain long-term self-renewal in vitro have been successfully derived from mouse embryos. Mouse embryonic stem (ES) cells can be derived from preimplantation EPI using serum or serum-free medium (Evans and Kaufman, 1981; Martin, 1981; Ying et al., 2008). In chimeric experiments, ES cells readily contributed to fetal tissues but rarely to extraembryonic tissues (Beddington and Robertson, 1989). The EPI after implantation can be used to establish primed embryonic stem cell line epiblast stem cells (EpiSCs), which are unable to colonize the embryo (Brons et al., 2007). Both preimplantation TE and postimplantation ExE can be used to derive trophoblast stem (TS) cells (Tanaka et al., 1998; Hayakawa et al., 2015). Extraembryonic endoderm (XEN) stem cells cannot only be established from PrE but also can be induced from ES cells due to the close relationship between EPI and PrE (Kunath et al., 2005; Cho et al., 2012; Niakan et al., 2013). TS and XEN cells contribute only to extraembryonic tissue *in vivo*.

In recent years, some totipotent-like stem cells have been found to contribute to both embryonic and extraembryonic tissues. Some of these cells exist for only a short period and are not sustained over the long term (Abad et al., 2013; Morgani et al., 2013; Macfarlan et al., 2012). In 2017, two stable totipotent-like stem cell lines were established (extended pluripotent stem [EPS] cells and expanded potential stem cells [EPSCs]) using serum-free culture conditions with different small molecule inhibitors (LCDM and JXSA) (Yang et al., 2017a; Yang et al., 2017b). However, their extraembryonic development ability remains controversial (Posfai et a, 2021). Recently, three new types of totipotent-like stem cell that can express 2-cell-specific genes under chemical-induced medium has been reported, but their extraembryonic tissue development efficiency needs to be further verified (Shen et al., 2021; Xu et al., 2022; Yang et al., 2022).

Using embryonic stem cells to de novo assemble the embryonic structure has become a powerful tool to study embryonic development. Embryoid bodies (EBs) are the original embryonic-like structure generated by the self-assembly and random differentiation of ES cells (Doetschman et al., 1985; ten Berge et al., 2008; Fuchs et al., 2012). The greatest disadvantage of this 3D structure is disordered tissues. On this basis, by inducing certain signaling pathways or adding extracellular matrix, the gene expression or tissue formation of embryos during postimplantation development can be simulated (van den Brink et al., 2014; Poh et al., 2014; Turner et al., 2017; Beccari et al., 2018; van den Brink et al., 2020; Moris et al., 2020; Veenvliet et al., 2020). In recent years, several research groups have used different combinations of ES, TS, XEN and EPS cells to construct embryo-like structures with certain characteristics of preimplantation or postimplantation embryos in vitro by self-assembly or self-organization (Harrison et al., 2017; Rivron et al., 2018; Sozen et al., 2018; Sozen et al., 2019; Zhang et al., 2019; Amadei et al., 2021). Interestingly, blastocyst-like structures (EPS-blastoids) with TE, EPI, and PrE lineage were constructed using only EPS cells. However, these structures do not develop into normal postimplantation embryonic structures in vivo or in vitro despite manifesting a similar cell lineage, cystic structure, and embryonic polarity to blastocysts (Li et al., 2019). The nature of the mechanism of EPS-blastoids formation and its relationship with developmental arrest remain unknown.

In this study, we revealed that EPS-blastoids had defects in the TE lineage differentiation. Through comparative analysis, we identified a small group of specific PrE-like cells pre-existing in EPS cell lines. And this PrE differentiation tendency is essential for blastoid formation with a “TE-like structure” (the outer layer of EPS-blastoids). The absence of real TE is the main reason for EPS-blastoid failure in normal implantation into the uterus and development into EPC and ExE. More importantly, we demonstrate that blastocysts reconstructed by the aggregated structure of pre-induction EPS cells (bilineage embryo-like structure [BLES]) and tetraploid blastocyst or tetraploid TE could implant normally and develop into live fetuses.

## Results

### EPS-blastoids show developmental defects in the TE lineage

**Figure 1.**
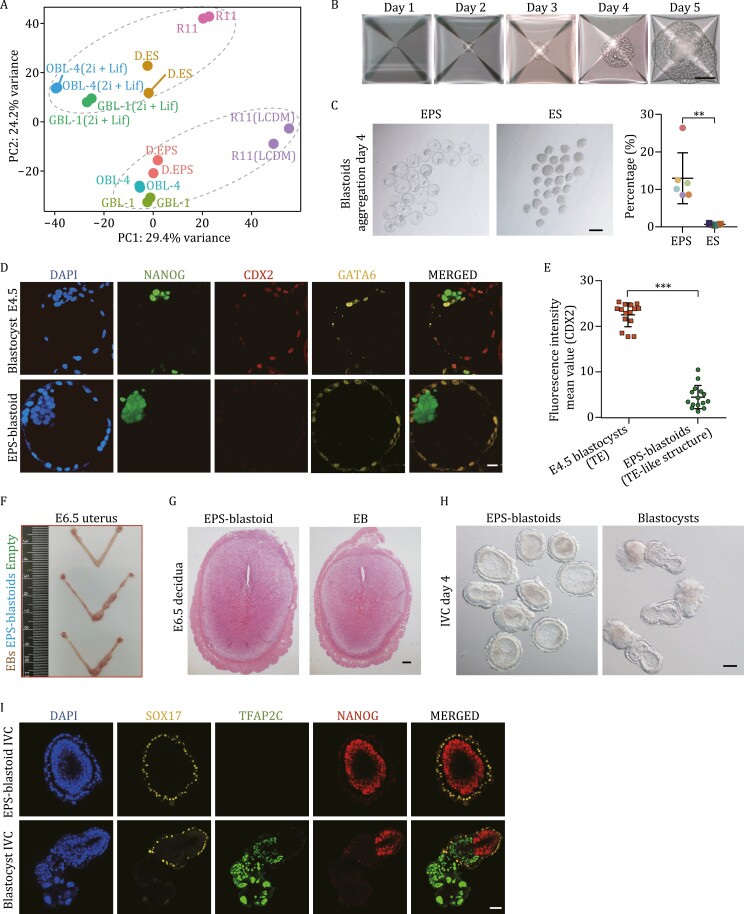
EPS-blastoids had developmental defects in the TE lineage. (A) PCA of bulk RNA-seq data of multiple EPS cell lines and ES cell lines from this study and published bulk RNA-seq data. EPS cell lines: D. EPS (Yang et al., 2017b), R11 (LCDM) (this study, converted from R11), GBL-1 (this study, derived from blastocyst), OBL-4 (this study, derived from blastocyst). ES cell lines: R11 (this study, derived from blastocyst), GBL-1 (2i/LIF) (this study, converted from GBL-1), OBL-4 (2i/LIF) (this study, converted from OBL-4), D. ES (Yang et al., 2017b). (B) Representative morphological images of EPS-blastoids at the indicated time point during the formation process. Scale bar, 100 μm. (C) Representative morphological images of EPS cells and ES cells on the 4^th^ day of aggregation (left) and the proportion of blastoid formation with different EPS and ES cell lines (right). *n* = 6 EPS cell lines were assayed, *n* = 5 ES cell lines were assayed. Data are represented as the mean ± SD; **P < 0.01, unpaired Student’s *t*-test. Scale bar, 200 μm. (D) Immunofluorescence staining of E4.5 embryos (top) and EPS-blastoids (bottom). Staining for NANOG (EPI), CDX2 (TE), and GATA6 (PrE). Scale bar, 20 μm. (E) Fluorescence intensity mean value (CDX2) of each E4.5 embryo’s TE and each EPS-blastoid’s TE-like structure. *n* = 15 E4.5 embryos were assayed, *n* = 15 EPS-blastoids were assayed. Data are represented as the mean ± SD; ***P < 0.001, unpaired Student’s *t*-test. (F) Representative images of the E6.5 uterus after transfer with EPS-blastoids or EBs at 3.5 dpc. (G) Histological analyses showing the E6.5 decidua induced by EPS-blastoids and EBs. Scale bar, 200 μm. (H) Brightfield image of postimplantation structures of EPS-blastoids and blastocysts cultured with an *in vitro* culture (IVC) system for 4 days. Scale bar, 100 μm. (I) Immunofluorescence staining of postimplantation structures of EPS blastoids and blastocysts cultured with an in vitro culture (IVC) system for 4 days. Staining for NANOG (EPI), TFAP2C (ExE and EPC), and SOX17 (VE). Scale bar, 50 μm. See also Fig. S1.

To examine the developmental potential of EPS-blastoids, we first established several EPS cell lines through derivation from 4-cell embryos to blastocysts or conversion from mouse ES cells (2i/LIF) using published method (LCDM) (Yang et al., 2017b). These EPS cell lines can maintain a stable morphology (Fig. S1A), which is similar to that of Deng-EPS cells (Yang et al., 2017b). Principal component analysis (PCA) of bulk RNA-seq also showed that both the derived EPS cells (GBL-1 and OBL-4) and converted EPS cells [R11 (LCDM)] that we established had a similar overall gene expression pattern as Deng-EPS cells (D. EPS) (Fig. 1A). The tetraploid complementation experiment showed that these EPS cell lines had good cell activity and pluripotency to produce fertile mice (Fig. S1B).

Next, we successfully cultured EPS-blastoids following previously reported methods (Li et al., 2019) using EPS cells (Fig. 1B). The diameter of EPS-blastoids reached approximately 100 μm after aggregation for approximately 4 days, which is similar to that of expanded blastocysts (Fig. 1B). In contrast to that of blastocysts, the diameter of EPS-blastoids exceeded 100 μm with continued culture (Fig. 1B). The formation efficiency of EPS-blastoids varied among different EPS cell lines (7%–27%) (Fig. 1C). In contrast, ES cells had a limited capability (less than 1%) to form blastoids, and most of the cells could only form solid EBs on Day 4 (Fig. 1C). Moreover, we detected the marker genes of each cell lineage through immunofluorescence staining experiments to compare EPS-blastoids with expanded blastocysts (Figs. 1D, S1C and S1D). In EPS-blastoids, we detected CDX2 signals in the external TE-like structure and NANOG signals in the internal ICM-like structure (Fig. S1C). However, CDX2 signals in TE of E4.5 embryos were much higher than that in TE-like structure of EPS-blastoids (Fig. 1D and 1E). In addition, some ICM-like structures of EPS-blastoids also expressed CDX2 (Fig. S1D). Besides, a large number of cells expressing the PrE lineage markers GATA6, SOX17, and PDGFRα were detected in the TE-like structure (Figs. 1D and S1D). The cellular composition of EPS-blastoids has been confirmed with different genetic backgrounds cell lines including TT2-6 obtained from Deng Lab and they showed consistent proportion (Fig. S1E and Fig. S1F). These results suggest that the TE-like structure of EPS-blastoids differs from the TE of normal blastocysts.

To evaluate the developmental potential after implantation, EPS-blastoids produced by EPS cells and EBs produced by ES cells, both cultured in EPS-blastoid medium, were transplanted into the uteri of pseudopregnant female mice. To our surprise, both entities induced decidual responses at E6.5 and showed similar decidualization rates (Figs. 1F and S1G). The decidua induced by EPS-blastoids contained no normal embryonic tissue, although it was larger than that induced by the EBs (Figs. 1G and S1H). We also compared the postimplantation developmental potential of EPS-blastoids and blastocysts using in vitro embryo culture (IVC) methods (Ma et al., 2019). After 4 days of culture, the cell lineage marker genes of these two products were detected by immunofluorescence (Fig. 1H and 1I). The results showed that blastocysts developed an E5.5-like morphology containing EPI, VE, ExE, and EPC, while EPS-blastoids developed lumen-like structure most containing only VE-like and EPI-like structures (Figs. 1H, 1I and S1I). In sum, we found that although EPS cells (LCDM condition) could successfully self-organized into EPS-blastoids, this blastocyst-like structure could not achieve normal embryonic development either *in vivo* or *in vitro*, especially in the absence of the TE lineage.

### The TE-like structure of EPS-blastoids is more similar to PrE-related cells of blastocysts than TE

**Figure 2.**
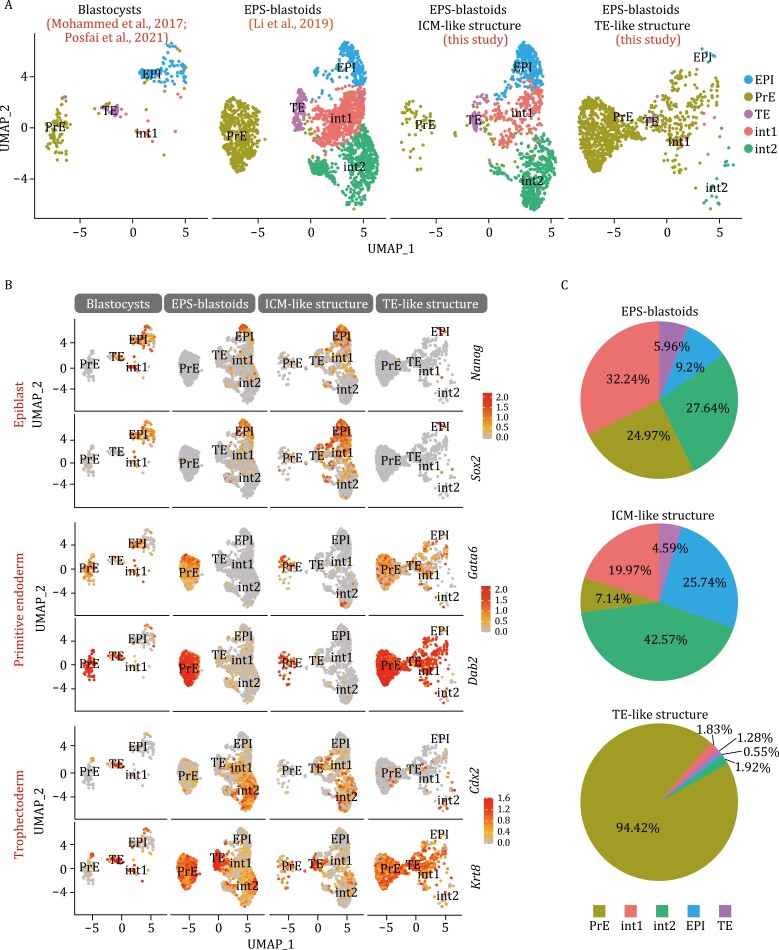
The TE-like structure of EPS-blastoids is more similar to PrE-related cells of blastocysts than TE. (A) Uniform manifold approximation and projection (UMAP) embedding of single-cell transcriptomes from EPS blastoids (Li et al., 2019), blastocysts (E3.5 and E4.5 embryos) (Mohammed et al., 2017, Posfai et al., 2021) and TE-like structures and ICM-like structures of EPS-blastoids (this study). EPI: Epiblast, PrE: Primitive endoderm, TE: Trophectoderm, int-1: intermediate state 1, int-2: intermediate state 2. (B) UMAP embedding of EPS blastoids and blastocysts. Shown is the expression of three lineage markers (EPI, *Nanog* and *Sox2*; PrE, *Gata6* and *Dab2*; TE, *Cdx2* and *Krt8*). (C) Percentage of cells in each cluster derived from EPS blastoids (Yang et al. 2017b), ICM-like and TE-like structures (this study). See also Fig. S2.

Although the TE-like structure of EPS-blastoids was morphologically very similar to the TE of blastocysts, an obvious deficiency in implantation and TE-related differentiation was found (Figs. 1F–I and S1G–I). To determine the identity of the TE-like structure, we separated the TE-like structure from the ICM-like structure using an enzyme-assisted microdissection method and then applied single cell RNA-seq to obtain their transcriptome individually. Next, the single-cell transcriptome data (1,094 cells for TE-like structure and 1,177 cells for ICM-like structure) were integrated with published single-cell transcriptomes derived from EPS-blastoids (Li et al., 2019) and blastocysts (E3.5 and E4.5) (Mohammed et al., 2017; Posfai et al., 2021). Integrated analysis using SEURAT revealed that the cells from published EPS-blastoids and our dissociated ICM-like and TE-like structures largely overlapped each other, suggesting that transcriptome of our blastoids is similar to that of published blastoid (Fig. 2A). Clustering analysis divided all cells into five clusters, including TE, ICM/EPI, and PrE which were shared by both EPS-blastoids and blastocysts, and two intermediate states mostly existed in EPS-blastoids, which is consistent with previous reports (Li et al., 2019) (Fig. 2A and 2B).

Surprisingly, we found that 94.42% of the cells in our isolated TE-like structure were clustered with PrE related cells, while PrE related cells only accounted for 7.14% in ICM-like structure (Fig. 2C). In published single cell data, the ratio of PrE related cells was 24.97% (Fig. 2C). These PrE-related cells specifically expressed PrE lineage marker genes (*Gata6*, *Gata4*, *Dab2*, *Pdgfrα*, and *Sox17*) (Figs. 2BS2B and S2D; Table S1). Notably, TE-related cells were mainly distributed in ICM-like structure rather than TE-like structure (Fig. 2A–C). Moreover, these cells did not typically express *Cdx2* at high levels; on the contrary, the cells with high expression of *Cdx2* are concentrated in the intermediate state 2 cluster, which was consistent with public data (Fig. 2B). It is worth noting that *Krt8* and *Krt18*, two TE markers, were expressed in PrE cluster and TE cluster simultaneously in our and public single cell data (Figs. 2B and S2C).

Therefore, we conclude that the TE-like structure of EPS-blastoids was mainly composed of PrE-related cells and the ICM-like structure was a mixture of several cell lineages. TE-related cells mainly exist in ICM-like structure and are not typical TE cells.

### PrE-related cells surrounded the ICM-like structure and expanded the cavity in the process of EPS-blastoid formation

**Figure 3.**
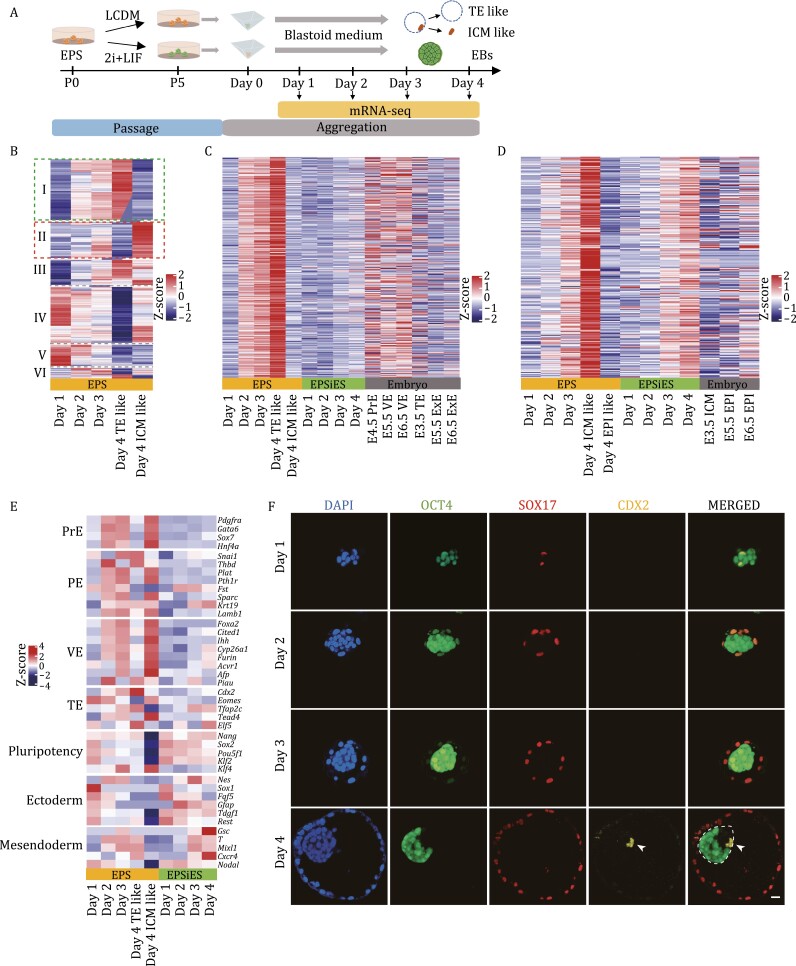
PrE-related cells surrounded the ICM-like structure and expanded the cavity in the process of EPS-blastoids formation. (A) Schematic of cell treatment, blastoid aggregation and mRNA-seq. EPS cells were cultured for 5 passages under LCDM or 2i/LIF conditions and then aggregated into EPS-blastoids or EBs with EPS-blastoids medium. RNA was collected at the indicated time point. EPS-blastoids on Day 4 were divided into TE-like structures and ICM-like structures, and RNA was collected. (B) Six groups of dynamically expressed genes during the formation of EPS-blastoids. (C and D) The relative expression of genes upregulated in TE-like structures (C) and ICM-like structures (D) in the process of EPS blastoid and EB formation and embryonic tissues. (E) Heatmap showing the relative expression of the indicated lineage-specific genes during the formation of EPS-blastoids and EBs. (F) Immunofluorescence staining of the indicated lineage marker genes during the formation of EPS-blastoids. Staining for OCT4, CDX2, and SOX17. The white dotted line indicates the area of the ICM-like structure, and the white triangles indicate cells expressing CDX2. Scale bar, 20 μm. See also Fig. S3.

To explore the formation mechanism of the TE-like structure, we designed experiments to obtain the transcriptome in the process of EPS-blastoid aggregation with SMART-seq2 methods (Fig. 3A). Aggregated EPS cell products were collected every 24 h for transcriptome sequencing during blastoids aggregation. EPS-blastoids on the fourth day were divided into two parts: TE-like structures and ICM-like structures (Fig. 3A). At the same time, ES cells (EPSiES) converted from EPS cells cultured for 5 passages in 2i/LIF medium served as a control to reduce the differences among different genetic backgrounds (Fig. 3A). The aggregated products were collected under the same conditions as EPS-blastoids for transcriptome sequencing.

Comparison of transcriptomes for different time points during blastoid formation revealed a large number of dynamically expressed genes. These dynamically expressed genes could be classified into six groups in chronological order (Fig. 3B). Among them, the genes of Group I and Group II were specifically upregulated during the formation of TE-like structures and ICM-like structures, respectively (Fig. 3B). After further analysis, we found that the genes upregulated in the TE-like structure (Group I) exhibited low expression level in embryonic TE-related lineages and EBs but were upregulated in embryonic PrE-related lineages (Fig. 3C and Table S2). Gene ontology (GO) analysis showed that the upregulated genes of the TE-like structure were enriched in nutrition transport, hormone secretion and transport, lipid metabolism and other pathways (Fig. S3A and Table S2). The expression of Group II genes showed similar trends during the formation of ICM-like structures and EBs (Fig. 3D and Table S2). In contrast, these genes were expressed at low levels in embryonic EPI-related cell lineages (Fig. 3D and Table S2). GO analysis showed that the upregulated genes of ICM-like structures were enriched in multiple developmental pathways, such as embryonic organ development and morphogenesis, anterior/posterior pattern specification, and skeletal system morphogenesis (Fig. S3B and Table S2), indicating that the ICM-like structure of EPS-blastoids was in a multiple-cell type mixing state similar to EBs. We further explored cell lineage marker gene expression in blastoid formation. The results clearly demonstrated that the formation of TE-like structures was actually due to differentiation of PrE-related cell lineages (Fig. 3E).

Moreover, proteins related to pluripotency and differentiation (PrE and TE) were detected during the formation of EPS-blastoids by immunofluorescence and the proportion of different types of blastoid was also calculated (Figs. 3F and S3C). On the first day, all aggregated products expressed the pluripotency marker OCT4, and in some products SOX17 (a marker of PrE) was detected in a small number of cells (Figs. 3F and S3C). In about half of the products, OCT4^+^SOX17^+^ cells gradually increased and wrapped around the OCT4^+^SOX17^−^ cells from Day 1 to Day 2 (Figs. 3F and S3C). Although the proportion of OCT4^+^SOX17^+^ cells were gradually increased in aggregated products at Day 3, only a small number of the products (7%–27%) would further expand outward to form a cavity (Figs. 1C, 3F and S3C). In these products, OCT4^+^SOX17^+^ cells gradually downregulated the expression of OCT4, expanded outward, and formed the TE-like structure of blastoid with a diameter of approximately 100 μm at Day 4 (Fig. 3F). As mentioned in Figs. 2, S1D and S1F, the ICM-like structure of some blastoids differentiated into several different cell types during the formation process: some cells no longer expressed OCT4, and some cells began to express CDX2 (Figs. 3F, S1D and S1F). Our results confirmed that EPS-blastoids did not have a real TE lineage but were a vesicular structure formed with differentiated PrE-related cells.

### LCDM medium can induce the emergence of PrE-like cells

**Figure 4.**
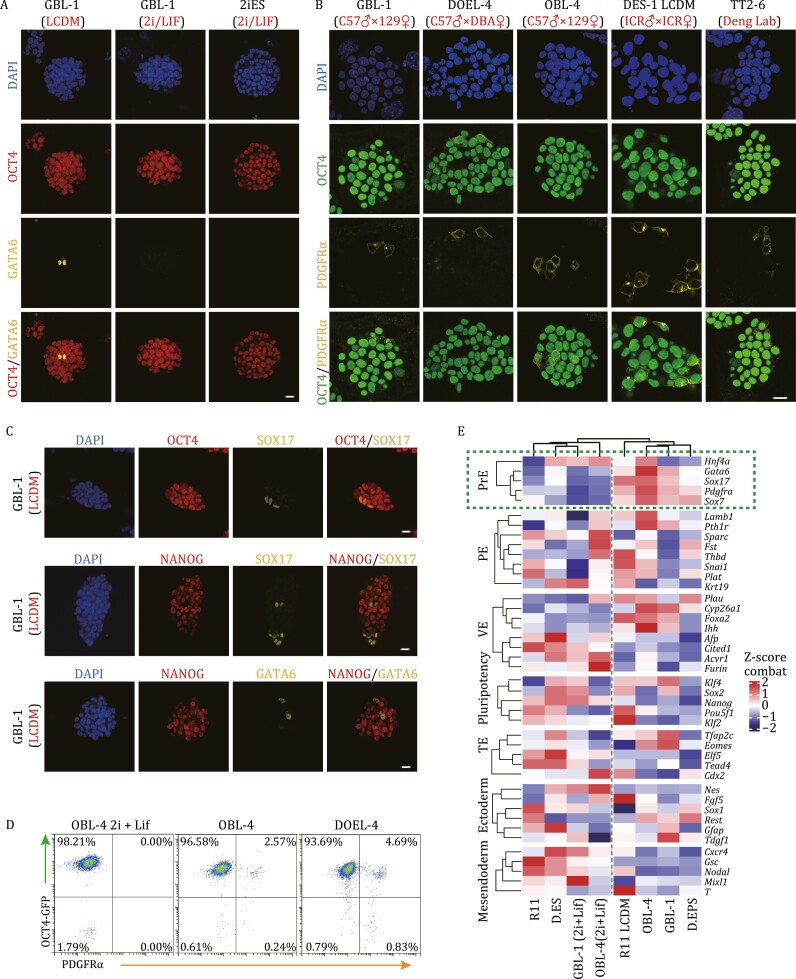
LCDM medium can induce the emergence of PrE-like cells. (A, B, and C) Immunofluorescence staining of EPS cells and ES cells. EPS cells: GBL-1, DOEL-4, OBL-4, DES-1 LCDM, and TT2-6 (Deng Lab) cells under LCDM medium. Converted ES cells: GBL-1 cells cultured with 2i/LIF medium; Derived ES cells: 2iES cells cultured with 2i/LIF medium. Staining for OCT4 and GATA6 (A), OCT4 and PDGFRα (B), and OCT4, NANOG, SOX17, and GATA6(C). Scale bars, 20 μm. (D) Representative FACS analysis of the percentages of OCT4^+^ PDGFRα^+^ cells in ES cell line (OBL-4 2i+Lif) and EPS cell lines (OBL-4 and DOEL-4). (E) Heatmap showing the relative expression of the indicated lineage-specific markers in EPS cell lines [GBL-1, OBL-4, R11 LCDM, and D. EPS (Yang et al., 2017b)] and ES cell lines [GBL-1 [2i+Lif], OBL-4 [2i+Lif], R11 and D. ES (Yang et al., 2017b)]. See also Fig. S4.

According to our previous experiments, EPS cells more easily differentiate into the PrE-related cell lineages during the formation of EPS-blastoids than ES cells. The difference in blastoid formation efficiency between EPS cells and ES cell lines prompted us to explore whether a subpopulation characterized by PrE lineage differentiation exists in EPS cells. To confirm this hypothesis, immunofluorescence staining was performed on EPS cells and ES cells. A small number of cells expressing PrE marker proteins (GATA6, SOX17, and PDGFRα) were detected in EPS cell clones under LCDM conditions, and these cells also expressed the pluripotency protein OCT4 (Fig. 4A–C). This suggests the presence of PrE-like cells in EPS cell lines with different genetic backgrounds, including cell line TT2-6 from Deng Lab. ES cells could only express the pluripotency protein OCT4 but not the PrE marker proteins GATA6 whether they were derived under 2i/LIF conditions or converted from EPS cells under 2i/LIF conditions (Fig. 4A). Similar to the PrE of the E4.5 blastocyst, these GATA6^+^ and SOX17^+^ cells in EPS cell cultures colocalized with OCT4 but not with NANOG (Fig. 4C). To further verify the proportion of PrE-like cells, PDGFRα staining and FACS (fluorescence-activated cell sorting) were used in different EPS cell lines. In cell lines with OCT4-GFP reporter, the percentage of OCT4^+^ PDGFRα^+^ cells were about 2%–5% (Fig. 4D). The proportion of PDGFRα^+^ in other cell lines (without OCT4-GFP reporter) is about 2% to 6% (Fig. S4A). Moreover, transcriptome analysis showed that early PrE-related genes were more highly expressed in EPS cells than in ES cells and converted ES cells under 2i/LIF conditions (Fig. 4E). Although PE and VE are differentiated from PrE after implantation, the PE- and VE-related genes showed no significant bias between EPS, ES and converted ES cells (Fig. 4E). These results indicated that EPS cells under LCDM conditions can autonomically produce GATA6/SOX17/PDGFRα^+^OCT4^+^ PrE-like cells and the PrE-like cells represent an early differentiation stage of the PrE lineage.

### *Gata6*-dependent PrE-like cells are essential for the formation of EPS-blastoids

**Figure 5.**
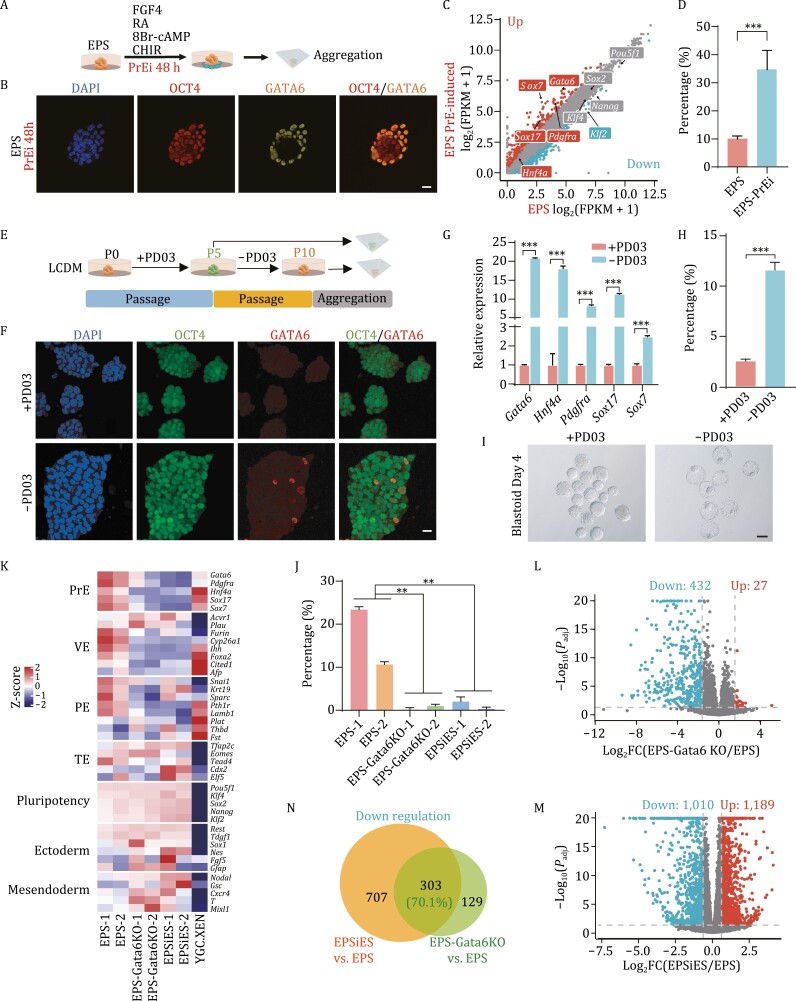
*Gata6*-dependent PrE-like cells are essential for the formation of EPS-blastoids. (A) Schematic showing the process of EPS-blastoids aggregation from EPS cells treated with PrE induction medium (N2B27 supplemented with FGF4, retinoic acid, 8Br-cAMP and CHIR) for 48 h. (B) Immunofluorescence staining of EPS cell clones after treatment with PrE induction medium for 48 h. Staining for OCT4 and GATA6. Scale bar, 20 μm. (C) Scatterplots showing differentially expressed genes in EPS cells after PrE induction medium treatment. (D) Quantification of EPS-blastoids formation efficiency in EPS cells before and after PrE induction medium treatment. Data are represented as the mean ± SD; ***P < 0.001, unpaired Student’s *t*-test. (E) Schematic showing EPS cells treated with the MEK inhibitor PD0325901. EPS cells were cultured under PD03 treatment for 5 passages and then divided into two parts. One part was cultured for another 5 passages after PD03 was removed, and the other part maintained the addition of PD03. Finally, EPS-blastoids were constructed with these two cell lines. (F) Immunofluorescence staining of EPS cell clones with or without PD03 addition. Staining for OCT4 and GATA6. Scale bar, 20 μm. (G) Relative expression of PrE lineage-specific genes in EPS cells with or without PD03 addition by qRT-PCR. Data are represented as the mean ± SD; ***P < 0.001, unpaired Student’s *t*-test. (H and I) Quantification of EPS-blastoids formation efficiency in EPS cells with or without PD03 (H) and representative morphological images of products aggregated by cells under these two conditions (I). Data are represented as the mean ± SD; ***P < 0.001, unpaired Student’s *t*-test. Scale bar, 100 μm. (J) Quantification of EPS-blastoid formation efficiency in the indicated cell lines. Data are represented as the mean ± SD; **P < 0.01, unpaired Student’s *t*-test. (K) Heatmap showing the relative expression of the indicated lineage-specific genes in four types of cell lines. EPS cells: EPS-1 and EPS-2 (this study). EPS cells treated with *Gata6*-KO: EPS-Gata6KO-1 and EPS-Gata6KO-2 (this study). ES cells converted from EPS cells: EPSiES-1 and EPSiES-2 (this study). XEN cells: YGC. XEN (Zhong et al., 2018). (L and M) Scatterplots of differentially expressed genes in EPS-Gata6KO cell lines (L) or EPSiES cell lines (M) relative to EPS cell lines from mRNA-seq data. (N) Venn diagram for the number of shared downregulated genes between the indicated groups. See also Fig. S5.

To confirm whether the formation efficiency of EPS-blastoids was improved when the proportion of PrE-like cells was increased in EPS cells. Therefore, we treated EPS cells with previously reported PrE induction medium (FGF4, CHIR, 8Br-cAMP, and RA) (Vrij et al., 2019) for 48 h and then aggregated them in EPS-blastoids medium for 4 days (Fig. 5A). GATA6/PDGFRα^+^OCT4^+^ cells were significantly increased more than eight times after treatment with PrE induction medium, and PrE marker genes were significantly upregulated at the transcriptional level (Figs. 5B, 5C and S5A; Table S3). As expected, more OCT4^+^SOX17^+^ structures were generated during the formation of the aggregated products, and finally the formation efficiency of EPS-blastoids increased threefold (Figs. 5D and S5B). These PrEi EPS-blastoids and their IVC culture products were immunofluorescent stained and showed consistent characteristics with normal EPS-blastoids (Fig. S5C and S5D). In addition, blastoids could also be successfully formed with ES cells after treatment with PrE induction medium (Fig. S5E and S5F).

In contrast, we speculate that if the differentiation of EPS cells into PrE was inhibited, EPS-blastoids formation would be prevented. To verify this hypothesis, we added PD0325901 (hereinafter referred to PD03) to LCDM medium (Fig. 5E), which has been reported to inhibit the specification of PrE by inhibiting the MEK signaling pathway in blastocysts (Nichols et al., 2009; Yamanaka et al., 2010). After 5 passages, GATA6^+^ cells were indeed significantly reduced in EPS cells (Fig. 5F). When PD03 was removed for another 5 passages, GATA6^+^ cells appeared again in EPS cells (Fig. 5F). In terms of gene expression, the expression of PrE-related genes in EPS cells with PD03 was significantly reduced compared with that in cells without PD03 (Fig. 5G). As expected, EPS cells supplemented with PD03 hardly formed EPS blastoids (Fig. 5H and 5I). The results illustrate that LCDM medium can induce the differentiation of EPS cells into PrE lineages.

According to previous reports, *Gata6*^−/−^ embryos cannot specify PrE precursors (Bessonnard et al., 2014; Cai et al., 2008; Schrode et al., 2014). Therefore, we knocked out *Gata6* in EPS cells to suppress the appearance of PrE-like cells (Fig. S5G and S5H). Aggregation experiments demonstrated that two EPS-Gata6KO cell lines with different deletion fragments could not form blastoids, similar to the EPSiES cell lines (Fig. 5J). Furthermore, transcriptome analysis also showed that the PrE, PE, and VE genes were downregulated in EPS-Gata6KO cell lines compared with wild-type EPS cell lines (EPS-1 and EPS-2) (Fig. 5K). Notably, EPS-Gata6KO cell lines and EPSiES cell lines also showed similar gene expression for the markers of other lineages (TE, pluripotency, ectoderm, and mesendoderm lineages) (Fig. 5K). From the perspective of overall expression differences, 27 genes were upregulated and 432 genes were downregulated after *Gata6* knockout in EPS cell lines (Fig. 5L and Table S4). EPSiES cell lines had 1,189 upregulated genes and 1,010 downregulated genes compared with EPS cell lines (Fig. 5M and Table S4). EPS-Gata6KO cell lines and EPSiES cell lines had 303 overlapping genes in the downregulated cluster, accounting for more than 70% of the downregulated genes in EPS-Gata6KO cells (Fig. 5N). However, few overlapping upregulated genes were identified between EPS-Gata6KO cell lines and EPSiES cell lines (Fig. S5I). GO analysis showed that the overlapping downregulated genes were enriched in the ERK1 and ERK2 cascades, Wnt signaling pathway, and steroid metabolic process, which are important for PrE differentiation (Fig. S5J and Table S4). Therefore, these overlapping downregulated genes are important to EPS-blastoids formation and PrE-like cell differentiation. Epigenetic modifications play an important role in cell fate determination, and we therefore detected H3K9AC, H3K4me1, H3K4me3, H3K27me3, and H3K27AC in the above three types of cell lines, followed by ChIP-seq. The results showed that H3K27AC in EPS-Gata6KO and EPSiES cells had significant erasure in the transcription start site (TSS) of these overlapping downregulated genes, but no significant changes in other epigenetic modifications were observed (Fig. S5K and S5L). These results suggested that GATA6 promotes the differentiation of EPS cells toward the PrE by affecting the distribution of H3K27AC. The above experiments showed that PrE-like cells in EPS cell culture determined blastoid formation.

### Blastocysts reconstructed by the BLES and tetraploid TE could implant and develop into normal fetuses

**Figure 6.**
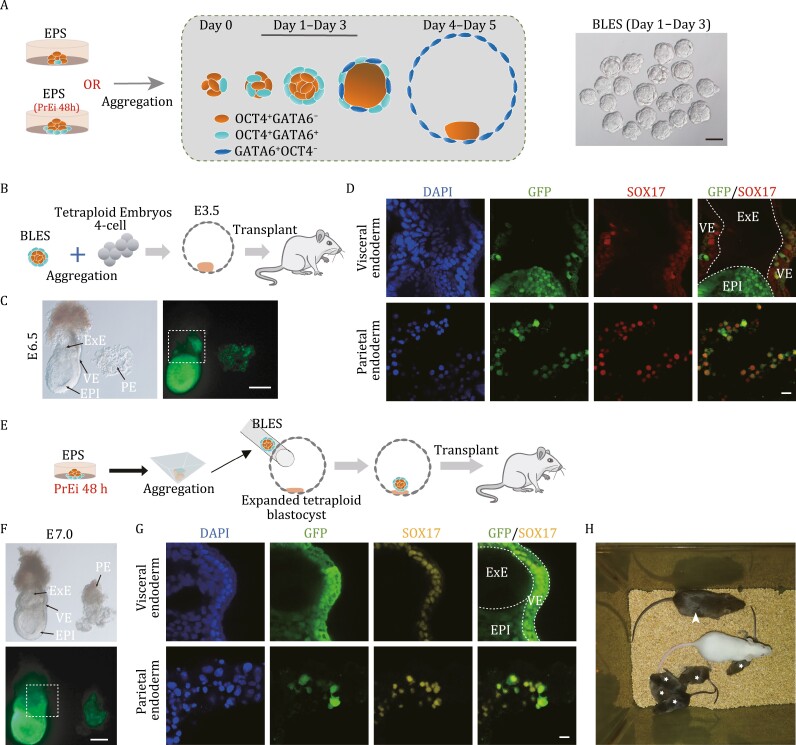
Blastocysts reconstructed by the BLES and tetraploid blastocysts could implant and develop into normal fetuses. (A) A diagram summarizing the formation process of EPS-blastoids (left). Representative images of BLES (right). Scale bar, 50 μm. (B) Schematic showing the procedure for embryonic chimeras. BLESs (produced by GFP-labeled EPS cells) at Days 2 and 3 were selected and aggregated with two tetraploid embryos at the “4-cell stage.” The chimeric embryos that developed into blastocysts were transplanted into the uteri of 2.5-dpc pseudopregnant mice. (C) Representative images of E6.5 chimeric embryos produced by the above method (B). The dotted area is also shown in 6D. Scale bar, 100 μm. (D) Immunofluorescence staining of E6.5 chimeric embryos produced by the above method (B). The top picture shows the dotted part in 6C. Staining for SOX17 (PE and VE). Scale bar, 20 μm. (E) Schematic showing the procedure for chimeras. BLESs were constructed using EPS cells treated with PrE induction medium for 48 h. Then, BLESs at Days 1 and 2 were selected and injected into tetraploid embryos at the expanded blastocyst stage. The chimeric embryos were transplanted into the uterus of a 2.5-dpc pseudopregnant mouse. (F) Representative images of E7.0 chimeric embryos produced by the above method (E). The dotted area is also shown in 6G. Scale bar, 100 μm. (G) Immunofluorescence staining of E7.0 chimeric embryos produced by the above method (E). The top picture shows the dotted part in 6F. Staining for SOX17 (PE and VE). Scale bar, 20 μm. (H) Brightfield image showing mice (arrow-marked) produced by the above method (E) and their offspring (asterisk) generated with ICR females. See also Fig. S6.

Initially implanted blastocysts (E3.5–E4.5) must have three layers of differentiation (TE, PrE, and EPI). We revealed the formation process of EPS-blastoids and found a small population of bilineage embryo-like structures (BLESs), which have obvious bilayer structures containing PrE and EPI formed between Day 1 and Day 3 (Fig. 6A). EPS-blastoids cannot develop into a normal postimplantation embryo *in vivo* or *in vitro* due to the lack of true TE in the outer layer. We hypothesized that blastocysts reconstructed by the BLES and tetraploid blastocysts could develop into live fetuses.

We first selected the BLES (Day 2–Day 3) constructed from GFP-labeled EPS cells for aggregation with tetraploid “4-cell embryos.” The aggregated blastocysts were then transplanted into the uteri of pseudopregnant mice (Fig. 6B). The results showed that the BLES successfully contributed simultaneously to the EPI, VE, and PE in E6.5 chimeric embryos (Fig. 6C and 6D).

Next, we wanted to verify whether supplementation with TE from blastocysts can support full-term development of BLES. Our previous experiments demonstrated that PrE-induced EPS cells were more effective for BLES and blastoid aggregation. Therefore, we directly injected the BLES (Day 1–Day 2) formed by PrE-induced EPS cells into the expanded cavity of tetraploid blastocysts (Fig. 6E). The reconstructed blastocysts were transplanted into the uteri of pseudopregnant female mice and showed normal embryo development (Fig. 6F–H). A large number of GFP^+^ cells emerged in the EPI, VE, and PE of the E7.0 chimera, which expressed the correct marker genes (Fig. 6F and 6G). The fetus, visceral yolk sac, and placenta of E12.5 chimera embryos also showed GFP^+^ cells (Fig. S6A). Immunofluorescent staining of E14.5 chimera placenta showed that GFP^+^ cells gathered in the labyrinth layer but not in the spongiotrophoblast layer (Fig. S6B). There was also obvious bilateral chimerism in the visceral yolk sac (Fig. S6C). The constructed embryos had good development potential. Six of 120 transferred constructed embryos were successfully grown until expiration. Furthermore, the BLES mice generated through BLES injection grew normally to adulthood and generated offspring by natural mating (Fig. 6H).

Moreover, we attempted to construct embryos using only the digested tetraploid TE with sheets and the BLES (Day 1–Day 2) (Fig. S6D). Although we obtained an E12.5 embryo with significant chimerism, no mice were ever born (Fig. S6E). These results demonstrated that the BLES has the ability to develop into live fetuses and all PrE-related lineages. Our exploration provides a feasible method for functional reconstructed embryos.

## Discussion

For a long time, research on early embryo development has been extremely difficult due to the scarcity of materials and inaccessibility. Therefore, in recent years, many researchers have been working to extend the in vitro embryo culture time or to use stem cells to simulate embryos. Several methods have been reported to simulate the structure of mouse and human preimplantation blastocysts, including the aggregation of multiple stem cell lines and self-organization of cells derived from the process of cellular pluripotent transformation (Fan et al., 2021; Kagawa et al., 2022; Kime et al., 2019; Li et al., 2019; Liu et al., 2021; Sozen et al., 2019; Sozen et al., 2021; Yanagida et al., 2021; Yu et al., 2021). However, at present, blastocyst-like structures cannot normally develop for long after implantation, posing a considerable challenge for their further application (Rossant and Tam, 2021). Therefore, because in vitro simulation of embryonic development has extremely important application prospects, we should assume a prudent attitude toward existing achievements.

In this study, we detected the developmental potential of EPS-blastoids and found that EPS-blastoids are not a three-lineage structure similar to blastocysts, and their TE-related lineage cannot be formed normally. Furthermore, through transcriptome analysis with location information, we found that the TE-like structure of EPS-blastoids was actually composed of PrE-related cells, although they expressed a small amount of *Cdx2*. Unsurprisingly, PrE-related cells can form cavities: PE and giant trophoblast cells (TGCs) form cavities called parietal yolk sacs after implantation *in vivo* (Rossant and Tam 2009); nEnd cells, which are closer to the primitive endoderm than XEN cells, can form a spherical cavity structure in a suspended state (Anderson et al., 2017). TE-like structure also expressed *Krt8* and *Krt18*, two TE marker genes. This atypical pattern of marker gene expression can indeed puzzled researchers. The same kind of confusion occurs in human blastocyst-like construction, and the TE-structure of iBlastoids generated through reprogramming of fibroblasts contains amnion-like cells according to recent reports (Zhao et al., 2021).

In addition, the further development potential of embryonic-like structures should be detected by both *in vivo* (embryo transfer) and *in vitro* (*in vitro* culture system) methods. Using an *in vivo* method, we found that both EPS-blastoids and EBs can induce the production of decidua without a normal embryo. However, the decidua can also be induced by abiotic substances such as mechanical trauma and oil. Therefore, the generation of decidua without a normal embryo could not prove the normal implantation of embryonic-like structures. Although in vitro culture can successfully prompt blastocyst development to the postimplantation stage, the resulting structures lack the normal morphology of PE and TGC, which play an important role in postimplantation development (enveloping the egg cylinder) (Bedzhov et al., 2014; Ma et al., 2019). Therefore, IVC is a limited method for evaluation of the postimplantation development of embryo-like structures. In general, verification of embryo-like implantation and postimplantation development is not easy.

We demonstrated that PrE-like cells were required for EPS-blastoids formation, which were initially present among EPS cells and were induced by LCDM culture conditions. EPS-blastoid medium could promote further proliferation of these PrE-early-differentiated cells. Similar to our study, a recent report proposed that EPS cells on LCDM medium were more likely to bifurcate to form EPI and PrE-like lineages in their blastoid medium than under LIF/serum conditions (Sozen et al., 2019).

Based on our results, we believed that the EPS-blastoids was not a blastocyst-like structure, so we named EPS-cystoids. Clearly, we came to a different conclusion from previous studies. The discrepancies may probably arise from that the blastoid generation are very sensitive to experimental parameters including clonal differences, culture medium, reagents and protocols. Although we tried to replicate the previous conditions as closely as possible, small experimental differences cannot be completely avoided. However, due to the requirement of extensive reproducibility, there is still room for improvement in this meaningful blastocyst model.

In this study, the early products (BLES) of EPS-blastoid (EPS-cystoids) aggregation were demonstrated to be able to develop into entire fetus and all PrE-derived lineages in the tetraploid complementary experiment. By adding functional TE-like cells with implantation abilities to BLES, achieving postimplantation development of cell-reconstructed embryos *in vivo* holds promise. Unfortunately, no cell lines have been found to be equivalent to embryo TE in the implantation area to date. Functional TE cell lines are urgently needed to construct embryonic-like models.

In a recent study, researchers achieved continuous mouse embryo culture from E5.5 to E11.0 outside of the uterus (Aguilera-Castrejon et al., 2021). This *in vitro* culture method, which does not require parietal yolk sac, suggests that intact gastrula development is feasible if high-quality E5.5 is reconstructed from cells. Therefore, 3D derivatives of EPS cells can be used as high-quality sources of EPI and VE for gastrula culture.

Taken together, we revealed that EPS-blastoids (EPS-cystoids) contain the PrE lineage and not the true TE lineage because EPS cells are prone to differentiate into PrE. We also demonstrate that the BLES has the potential to develop into EPI, PE, and VE. These structures can be further used to construct more ideal embryo-like structures.

## Materials and methods

### Animals

TgOG2 was a generous gift from Dr. Jeff R. Mann, University of Melbourne, Victoria, Australia (Szabo et al., 2002). B6.Cg-Tg(CAG-GFP)Smoc mice were obtained from Shanghai Model Organisms Center (China). ICR, 129, C57BL/6, and DBA2 mice were purchased from Charles River (China). All mice were housed in a specific pathogen-free (SPF) animal facility at Tongji University, Shanghai, China. F1 hybrids between B6.Cg-Tg(CAG-GFP)Smoc (male) and 129 (female), and F1 hybrids between TgOG2 (male) and 129 (female) mice were generated with 7- to 8-week-old females. ICR females aged 6–8 weeks were used as donors of tetraploid embryos and chimera embryos. ICR adult females aged 8–10 weeks were used as pseudopregnant recipients. All animal experiments and breeding procedures were performed in accordance with the experimental animal use guide of Tongji University.

### Culture of mouse stem cells

All mouse stem cells were maintained on mitomycin (MMC; Sigma-Aldrich, M0503)-treated mouse fibroblasts in a humidified incubator under 5% CO_2_ at 37°C, with the exception of a few experiments requiring feeder free. Cells were cultured with medium that was replaced every day or every other day, and at 80% confluence, the cells were passaged using 0.05% trypsin-EDTA and digested into single cells.

ES cells were cultured in ES medium (ESM) supplemented with LIF (10 ng/mL recombinant mouse LIF; Millipore, ESG1107) or 2i/LIF (1 µmol/L MEK inhibitor PD0325901 Selleck 1036S; 3 µmol/L GSK-3 inhibitor CHIR99021 Selleck S2924 and 10 ng/mL rmLIF). ESM contained DMEM (Sigma-Aldrich, D5671) supplemented with 15% (*v/v*) fetal bovine serum (FBS) (HyClone, SH30070.03), 1 mmol/L L-glutamine (Millipore, 25030-081), 0.1 mmol/L β-mercaptoethanol (GIBCO, 21985-023), 1× nonessential amino acid (Millipore, TSM-001-C), 1× penicillin-streptomycin (Thermo Fisher, 15140122), and 1× nucleosides (Millipore, ES-008-D).

EPS cells were cultured in N2B27 basal medium supplemented with 10 ng/mL recombinant human LIF (Millipore, LIF1050), 3 µmol/L CHIR99021, 2 µmol/L (S)-(+)-dimethylene maleate (Tocris, 1425), and 2 µmol/L minocycline hydrochloride (Selleck, s4226). N2B27 basal medium was composed of a 1:1 mixture of DMEM/F-12 (GIBCO, 11330-032) and neurobasal medium (GIBCO, 21103-049) supplemented with 0.5× N2 supplement (Invitrogen, 17502-048), 0.5× B27 supplement (Invitrogen 17504-044), 1× nonessential amino acid, 1 mmol/L L-glutamine, 0.1 mmol/L β-mercaptoethanol, and 5% KnockOut Serum Replacement (GIBCO 10828-028).

PrE induction medium was composed of N2B27 basal medium supplemented with 3 μmol/L CHIR99021, 100 ng/mL recombinant human FGF4 (PeproTech, 100-31), 10 nmol/L retinoic acid (Sigma, R2625), and 1 mmol/L 8Br-cAMP (Selleck, S7857).

TSC basal medium consisted of RPMI 1640 supplemented with 20% (*v/v*) FBS, 1× GlutaMAX (Thermo Fisher, 35050-061), 1× sodium pyruvate (Sigma-Aldrich, S8636), 0.1 mmol/L β-mercaptoethanol, and 1× penicillin-streptomycin.

### Culture of mouse embryos

Two-cell or blastocysts were obtained from the oviducts or uteri of mated females. All embryos were cultured using microdroplet culture methods with G-1 PLUS (Vitrolife 10128) medium covered with mineral oil in a humidified incubator with 5% CO_2_ at 37°C.

### Generation of cell lines

Both mouse ES cells and mouse EPS cells were derived from mouse 4-cell embryo to blastocysts as previously reported (Yang et al., 2017b). TT2-6 was a generous gift from Dr. HongKui. Deng, Peking University, Beijing.

Generation of *Gata6*-knockout EPS cell lines: five *Gata6* guide RNA sequences were cloned into the plasmid PX330 (Cong et al., 2013). Then, these 5 plasmids were cotransfected into EPS cells with nucleofection (4D-Nucleofector System, Lonza). These cells were seeded on feeders at a lower density (3,000 cells in 35-mm dishes). After the clones grew, the monoclones were selected and expanded individually. After genomic PCR identification, colonies with 43-bp deletion and 20-bp deletion of exon 2 of the *Gata6* locus were used for the next experiment.

### Generation of EPS-blastoids

The cell clones were first digested into single cells using 0.05% trypsin-EDTA and then transferred to a 0.1% gelatin-coated (Sigma, ES-006-B) plate for incubation for 15–30 min to attach the feeder cells to the plate. The cell suspension was collected, and 2,000–6,000 cells were placed in one well of a 24-well Aggrewell-400 (STEMCELL Technologies, 34415) plate cultured with EPS-blastoid basal medium supplemented with 2 µmol/L ROCK inhibitor Y-27632 (Selleck, S049), 12.5 ng/mL recombinant human FGF4, 0.5 µg/mL heparin (Sigma-Aldrich, H3149), 3 µmol/L CHIR99021, 5 ng/mL recombinant human BMP4 (PeproTech, 12-05ET), and 0.5 µmol/L A83-01 (Axon Medchem, 1421). EPS-blastoid basal medium consisted of 25% TSC basal medium, 25% (*v/v*) N2B27 basal medium, and 50% (*v/v*) KSOM (Aibei Biotechnology, M1430) or G-1 PlUS. The medium was replaced with fresh culture medium without Y-27632 the next day.

### IVC culture of blastocysts and blastoids

We followed previously reported methods (Ma et al., 2019). Blastocysts or blastoids were placed in 4-well plates coated with Matrigel (CORNING, 356234) and then cultured with IVC1 for 48 h, which was subsequently replaced with IVC2 for culture for another 48 h. IVC1 was composed of CMRL 1066 (Thermo Fisher Scientific 11530037) containing 10% (*v/v*) FBS, 1 mmol/L sodium pyruvate, 1× penicillin-streptomycin, 2 mmol/L L-glutamine, 1× N2 supplement, and 0.25× B27 supplement. IVC2 contained 20% (*v/v*) FBS, and the remaining components were the same as those in IVC1.

### Production of diploid or tetraploid embryos

ICR females aged 6–8 weeks were intraperitoneally injected with 5 IU of pregnant mare serum gonadotropin (PMSG) (San Sheng, China) and with 7 IU of human chorionic gonadotropin (hCG) (San Sheng) 48 h later. Superovulated ICR females were mated with 8- to 10-week-old ICR males, and vaginal plugs were assessed before noon on the next day (seemingly embryonic 0.5, E0.5). Embryos at the 2-cell stage (embryonic 1.5, E1.5) collected from the oviducts of mated ICR females were cultured using microdroplet methods with G-1 PLUS medium under mineral oil at 37°C and 5% CO_2_. Tetraploid embryos were produced with electrofusion at the late 2-cell stage. The tetraploid embryos were cultured in G-1 PLUS medium until aggregation.

### Aggregation of cells or the BLES and tetraploid embryos

#### For cells.

EPS cell colonies were treated with 0.05% trypsin-EDTA for approximately 4 min in a 37°C cell incubator to obtain a single-cell suspension. Then, the cell suspension was transferred to a 0.1% gelatin-coated plate and incubated for 15–30 min to attach the feeder cells to the plate. The collected cell suspension was placed on ice and set aside for use.

#### For BLES.

BLESs with obvious bilayer structure from Day 1 to Day 3 during blastoid formation were selected under an inverted microscope.

A group of 10–15 single cells or one BLES was transferred into depression wells of the aggregation plate. The tetraploid embryos were removed from the zone pellucida with 20 mg/mL PE (Pronase E; Sigma, #P8811) at the “4-cell stage,” and two embryos were used as a group located inside depression wells with cells or the BLES. The aggregated embryos were cultured into the blastocyst stage in G-1 PLUS medium and then transferred into the uteri of 2.5-dpc pseudopregnant recipients.

### Chimeras and microinjection of the BLES into tetraploid embryos

#### For cells.

Chimeras were produced by microinjection of 6–8 cells into normal diploid embryos at the 8-cell stage. The chimeras were cultured in G-1 PLUS medium until immunofluorescence analysis or transferred into the uteri of 2.5-dpc pseudopregnant recipients at the blastocyst stage.

#### For BLES.

One BLES with a bilayer structure collected on Days 1 and 2 was microinjected into the cavity of expanded blastocysts (E3.5–E4.25) of tetraploid embryos. The constructed embryos were recovered after 5 h and transferred into the uteri of 2.5-dpc pseudopregnant recipients.

### RT-qPCR

To analyze the gene expression of cells, total RNA was extracted by the phenol chloroform extraction method using 1 × 10^6^ cells. A cDNA library was synthesized using 5× All-In-One RT Master Mix (Abm, G490, Canada) with total RNA. Quantitative PCR (QPCR) was performed using Chamq Universal SYBR qPCR Master Mix (Vazyme Q711-02, China). Signals were captured and exported by a real-time PCR system (ABI7500, Applied BioSystems, USA). *Gapdh* and *β-actin* were used as endogenous controls to evaluate gene expression. The relative gene expression results were analyzed and exported by GraphPad Prism 7 software.

### Histological analyses

Decidua were fixed overnight with 4% paraformaldehyde (Servicebio, China) at 4°C. All the samples were embedded in paraffin and cut along the uteri, and 2–3 pieces were obtained. The sections were stained with hematoxylin and eosin (H&E) and then photographed with a microscope.

### Immunofluorescence

All cell clones and tissues were fixed with 4% paraformaldehyde (Servicebio, China) overnight at 4°C. After fixation, cell colonies, frozen sections, blastoids, and blastocysts were permeabilized with 0.3% Triton X-100 (Sigma-Aldrich, 93443) in DPBS (Gibco) for 30 min at room temperature. Then, the cells were blocked with 3% bovine serum albumin (BSA, MP Biomedicals) in DPBS for 1–2 h at room temperature. Postimplantation tissue of blastocysts or blastoids was permeabilized and blocked with 0.5% Triton X-100 in 3% BSA-DPBS for 2 h at room temperature. All samples were incubated with primary antibodies overnight at 4°C. All samples were then washed 3 times with DPBS containing 0.01% Triton X-100 and incubated with secondary antibodies in 3% BSA-DPBS at room temperature for 2 h. 4ʹ,6-Diamidino-2-phenylindole (DAPI) (Invitrogen D3571) was used to stain nuclei for 15–20 min at room temperature. The samples were washed with DPBS 3 times before imaging. All prepared samples were observed and photographed with a ZEISS LSM 880 confocal microscope. Simple image processing was performed using ZEISS processing software.

The primary antibodies and dilutions used were as follows: anti-OCT4 (1:200; Santa Cruz, sc-5279), mouse anti-CDX2 (1:400; Bio-Genex, MU392A-UC), rabbit anti-CDX2 (1:500; Abcam, ab76541), anti-NANOG (1:500; Reprocell, RCAB002P-F), anti-GATA6 (1:200; R&D Systems, AF1700), anti-SOX17 (1:200; R&D Systems, AF1924-SP), anti-PDGFRα (1:200; Abcam, ab203491), anti-TPBPA (1:500; Abcam, ab104401), and anti-TFAP2C (1:200; Santa Cruz, sc-12762). All secondary antibodies were from the Thermo Fisher’s Alexa Fluor series of antibodies, and all dilution ratios were 1:200.

### Western blot

Whole-cell protein extracts were isolated from EPS-1, EPS-2, EPS-Gata6KO-1, and EPS-Gata6KO-2 cell line using RIPA lysis buffer (Beyotime, P0013B) supplemented with protease inhibitor cocktail (Roche, 04693132001). Protein extracts were loaded onto SDS-polyacrylamide gel. Gel was run at 80 V for 30 min and 120 V for 1.5 h. Proteins were transferred for 2 h at 200 mA to a PVDF membrane (IPVH00010, Millipore). Blots were incubated in rapid blocking buffer (Epizyme Biomedical Technology, PS108) for 30 min. Primary antibodies were incubated overnight at 4°C, and secondary antibodies were incubated at RT for 1 h. The signals were measured using ECL reagents (GE) and visualized by the ChemiDoc MP imaging system (Bio-Rad).

The primary antibodies and dilutions used were as follows: anti-GATA6 (1:400; R&D Systems, AF1700), anti-β-Actin (1:2,000; Proteintech, HRP-66009). The primary antibodies and dilutions used were as follows: anti-goat IgG (H+L) (1:5,000; Beyotime, A0181), anti-mouse IgG (H+L) (1:5,000; Beyotime, A0216).

### Fluorescence-activated cell sorting

For FACS, cells were collected by 0.05% trypsin-EDTA digested for 4 min and washed with FACS buffer containing PBS supplemented with 2% FBS. After stained with anti-PDGFRα (1:50; Abcam, ab203491) antibodies, cells were washed and resuspended in FACS buffer. All analyses were performed on a MoFlo XDP cell sorter (MoFlo Astrios EQ).

### Smart-seq library

Single cells of blastoids or embryos were digested with 0.05% trypsin-EDTA and collected using mouth pipetting. All single cells were washed at least three times with 0.5% BSA-PBS solution before collection.

According to previous methods, approximately 20 cells were collected to compose each library (Picelli et al., 2014). Single cells were transferred into lysis buffer in a PCR tube by mouth pipetting. The samples were centrifuged (1,000 rpm, 3 min) at 4°C and immediately reverse transcribed or stored at −80°C. Reverse transcription was performed depending on the oligo (dT) primers in lysates. Second-strand cDNA was synthesized depending on template switching with a TSO. Fragmented cDNA was prepared for library construction with a Covaris sonicator (Covaris S220, Woburn, MA). Library construction was completed with a Kapa Hyper Prep Kit (KAPA, KK8504, Switzerland). The constructed libraries were sequenced on the Illumina NOVA platform with paired-end reads of 150 bp. Quality control and sequencing of constructed libraries were performed by Berry and Novogene Genomics Corporation.

### Bulk mRNA-seq library

Colonies of cells were digested with 0.05% trypsin-EDTA and collected with 1 mL TRIzol (Takara Bio, 9109). The samples were vortexed for 5 min and stored at −80°C or immediately submitted to RNA extraction. Total RNA was extracted with the phenol chloroform extraction method. A bulk mRNA-seq library was constructed with a KAPA Stranded mRNA-Seq Kit (KAPA, KK8421). The mRNA was captured by magnetic oligo-dT beads and fragmented by heating in the presence of Mg^2+^. The first strand was synthesized with random primers, and the second strand was synthesized by converting hybrid cDNA:RNA to dscDNA. The mRNA-seq library was sequenced on the Illumina NOVA platform with paired-end reads of 150 bp. Quality control and sequencing of construted libraries were performed by Novogene Genomics Corporation.

### Single-cell RNA-seq (scRNA-seq) library construction and sequencing

Around 400 EPS-blastoids were manually picked up. TE-like and ICM-like structure was separated using a mouth pipette with Acumax (Innovative Cell Tech, AM105) at 37°C for 10 min. The two structures were then digested again for 10 min with agitation. Dissociated cell suspensions were converted to barcoded scRNA-seq libraries using DNBelab C Series (MGI, 940-000047-00) through steps including droplet encapsulation, emulsion breakage, mRNA captured beads collection, reverse transcription, cDNA amplification, and purification. cDNA production was sheared to short fragments with 250–400 bp, and indexed sequencing libraries were constructed according to the manufacturer’s protocol. Qualification was performed using Qubit ssDNA Assay Kit (Thermo Fisher Scientific, Q10212) and Agilent Bioanalyzer 2100. All libraries were further sequenced by the MGISEQ-2000.

### ULI-NChIP-seq library

Colonies of cells were digested with 0.05% trypsin-EDTA and stored at 4°C for use. According to previous studies, 1,000 cells dispersed as single cells were used per library (Brind’Amour et al., 2015; Liu et al., 2016). Antibodies against histone H3K4me3 antibody (Cell Signaling Technology, 9727S), histone H3K27me3 antibody (Diagnode, C15410195), histone H3K27ac antibody (Active motif, 39133), histone H3K9ac antibody (Abcam, ab4441), or histone H3K4me1 antibody (Cell Signaling Technology, 5326s) at 1 µg were used for the immunoprecipitation reaction. Libraries were generated with a Kapa Hyper Prep Kit (KAPA, KK8504) and sequenced on the Illumina NOVA platform with paired-end reads of 150 bp. Quality control and sequencing were performed by Berry Genomics Corporation.

### Bulk RNA-seq data processing

Bulk RNA-seq data were first subjected to Trim_galore (version 0.6.4) for adaptor trimming as well as quality control with the parameters --paired -j 7 --basename. The trimmed paired-end reads were then aligned to the mm9 reference genome with random chromosomes cleaned by STAR (version 2.7.3a) (Dobin et al., 2013) under the parameters --runThreadN 30 --runMode alignReads --outSAMtype BAM SortedByCoordinate --outSAMstrandField intronMotif. Gene expression was quantified in FPKM (fragments per kilobase million) by Cufflinks (version 2.2.1) (Trapnell et al., 2010). For the downstream data analyses, FPKM values were averaged for each gene between replicates. The RefSeq gene annotation files were downloaded from UCSC. For genes with multiple isoforms, the longest transcripts were selected.

The R package DESeq2 (version 1.26.0) (Love et al., 2014) was used for gene differential expression analysis. A fold change > 2 and an FDR < 0.05 were used as cutoffs for downregulated and upregulated genes. Gene ontology (GO) analysis of genes was performed using the enrichGO function of the R package clusterProfiler (version 3.14.3) (Yu et al., 2012) under the parameters fun = “enrichGO”, pAdjustMethod = “BH”, ont = “BP”, OrgDb = org.Mm.e.g..db, keyType = “SYMBOL”, and qvalueCutoff = “0.05”. To perform PCA, FPKM values plus one from different datasets were log2-transformed before being subjected to batch effect removal by the R function ComBat from the package sva (version 3.34.0) (Jaffe et al., 2017), and then PCA values were calculated by the prcomp function from R’s built-in stats package.

The dynamic genes in Fig. 3B were identified by a strategy based on Shannon entropy described previously (Xie et al., 2013). Specifically, genes with low expression levels (average FPKM < 1) were excluded before calculating Shannon entropy. Shannon entropy was calculated by the entropy function of the R package DescTools (version 0.99.41). Based on examination of the entropy distribution, genes with entropy less than 2 were selected as the dynamically expressed genes. The FPKM plus one of these genes was then log2 transformed and finally subjected to Z-score scale. The Z-scores were then clustered by k-means (*n* = 15), and clusters with the same trends were merged. Expression heatmaps were constructed using Z-scores by the R package ComplexHeatmap (version 2.2.0) (Gu et al., 2016). In Fig. 4E, the batch effect between laboratory was removed using sva’s ComBat function, and the resulting normalized gene expression values were scaled to construct the figure.

Bulk RNA-seq data from published datasets were processed together with samples from this study. Published data were downloaded from the indicated GEO datasets: D. EPS (GSM2135530 and GSM2135531) and D. ES cells (GSM2135528 and GSM2135529) from GSE80732 (Yang et al., 2017b) and mouse XEN cells from GSE106158 (GSM2830587, GSM2830588, and GSM2830589) (Zhong et al., 2018). E4.5 PrE data from GSE100597 (GSM2687798, GSM2687802, GSM2687809, and GSM2687829) (Mohammed et al., 2017) cells were annotated by (Zhong and Binas 2019); E3.5 ICM and E3.5 TE data were from GSE168274 (under revision).

### Single-cell RNA-seq (scRNA-seq) analysis and integration

We integrated three scRNA-seq datasets: ICM-like structure and TE-like structure data generated by DNBelab C4 system in this study, 10× Genomics data of mouse EPS-blastoids (GSM4026211) were retrieved from GSE135701 (Li et al., 2019), and single-cell Smart-Seq2 data of mouse embryos E3.5 and E4.5 were downloaded from GSE100597 (Mohammed et al., 2017) and GSE145609 (Posfai et al., 2021). DNBelab C4 system scRNA-seq data were aligned following DNBelab_C_Series_HT_scRNA-analysis-software. The 10× Genomics single-cell data were mapped using STAR with parameters --soloType Droplet --soloCBstart 1 --soloCBlen 16 --soloUMIstart 17 --soloUMIlen 12 --soloStrand Unstranded --soloCBwhitelist. Raw read filtering and alignment of Smart-Seq2 data were performed as described for bulk RNA-seq processing. Then, the read counts per gene were summarized by the featureCounts function from the Subread package (version 2.0.0) (Liao et al., 2014).

After preprocessing, expression matrices were loaded into R (version 4.0.5) with Seurat (version 4.0.3) (Hao et al., 2021). Integration of 10× Genomics and Smart-Seq2 datasets was performed following Seurat’s integration pipeline. Cells with more than 5,000 UMIs and less than 25% of mitochondrial reads were included in the analysis. Genes detected in fewer than 10 cells across all single cells were filtered before normalization. Datasets were then scaled and log-transformed using the “NormalizeData” function. For integration, the 2,000 most-variable genes were identified by “FindVariableFeatures” with the following parameters: selection.method = “vst”, nfeatures = 2,000. Integration anchors were identified based on these genes using the canonical correlation analysis (CCA) integration tool with 30 dimensions as implemented in the “FindIntegrationAnchors” function. The data were then integrated using “IntegrateData” and scaled again using “ScaleData.” Principal component analysis (PCA) with 30 principal components was performed by “RunPCA,” and uniform manifold approximation and projection (UMAP) dimension reduction with 15 principal components was performed by “RunUMAP.” A nearest-neighbor graph using the 15 dimensions of the PCA reduction was calculated using “FindNeighbors,” followed by clustering using “FindClusters” with a resolution of 0.2. Seurat’s “DimPlot” was used to plot cell clusters, and the “FeaturePlot” function was used to demonstrate individual gene expression by UMAP embedding. Seurat’s “DotPlot()” was used to plot average expression for marker genes. FindConservedMarkers was used to identify marker genes which are conserved across different datasets for cell clusters. The top 50 markers were kept for each cluster to generate heatmap.

### ChIP-seq data analysis

ChIP-seq data were first subjected to Trim_galore (version 0.6.4) for adaptor trimming as well as quality control with the parameters --paired -j 7 --basename. The trimmed paired-end reads were then aligned to the mm9 reference genome with random chromosomes cleaned by bowtie2 (version 2.3.5.1) (Langmead and Salzberg, 2012) under the parameters --no-mixed --no-discordant. PCR artifacts were removed by MarkDuplicates of Picard Tools (version 2.21.1). Genome coverage bigwig files for aggregation plots were generated by the bamCoverage function of the Deeptools package (version 3.5.0) (Ramirez et al., 2016) with the parameters --normalize and RPKM -bs 50. Aggregation plots for different histone signals across genes were plotted by the computeMatrix and plotProfile functions of DeepTools.

## Supplementary Material

pwac029_suppl_Supplementary_Figures

pwac029_suppl_Supplementary_Table_S1

pwac029_suppl_Supplementary_Table_S2

pwac029_suppl_Supplementary_Table_S3

pwac029_suppl_Supplementary_Table_S4

## Abbreviations

BLES, bilineage embryo-like structure; EPI, epiblast; ExE, extraembryonic ectoderm; EPC, ectoplacental cone; GO, gene ontology; GFP, green fluorescent protein; H3K4me3, histone 3 lysine 4 trimethylation; H3K27me3, histone 3 lysine 27 trimethylation; H3K4me1, histone 3 lysine 4 monomethylation; H3K9ac, histone 3 lysine 9 acetylation; H3K27ac, histone 3 lysine 27 acetylation; ICM, Inner cell mass; IVC, in vitro embryo culture; KO knockout; LIF, leukemia inhibitory factor; PrE, primitive endoderm; PE, parietal endoderm; PCR, polymerase chain reaction; PCA, principal component analysis; TE, trophectoderm; VE, visceral endoderm.

## Declarations

The ULI-ChIP-Seq data, bulk mRNA-Seq data, Smart-seq data, and scRNA-seq data generated in this study have been deposited in the Genome Sequence Archive under project PRJCA006265. The accession number for the sequencing data reported in this paper is GSA: CRA004846. The code used in this project is provided at xiaocuixu/mouse_blastoidinGitHub.

Y.W., W.L. and S.G. conceived the project and provided mentoring; K.L., D.B. and W.L. designed and performed the experiments; X.X. provided bioinformatics support; Y.L., Y.Z., Y.J., M.G., Y.L., J.Y., X.H., Y.Z., X.K., Y.Z., S.L., J.C. and H.W. assisted with the experiments; K.L., D.B., W.L. and S.G. wrote the manuscript.
