# Supplementary material for: Bilineage embryo-like structure from EPS cells can produce live mice with tetraploid trophectoderm
Source: Protein Cell. 2022 Jul 15;14(4):262–78. doi: 10.1093/procel/pwac029 (PMC10120967; doi:10.1093/procel/pwac029)
Supplement: pwac029_suppl_Supplementary_Figures [file pwac029_suppl_supplementary_figures.pdf]

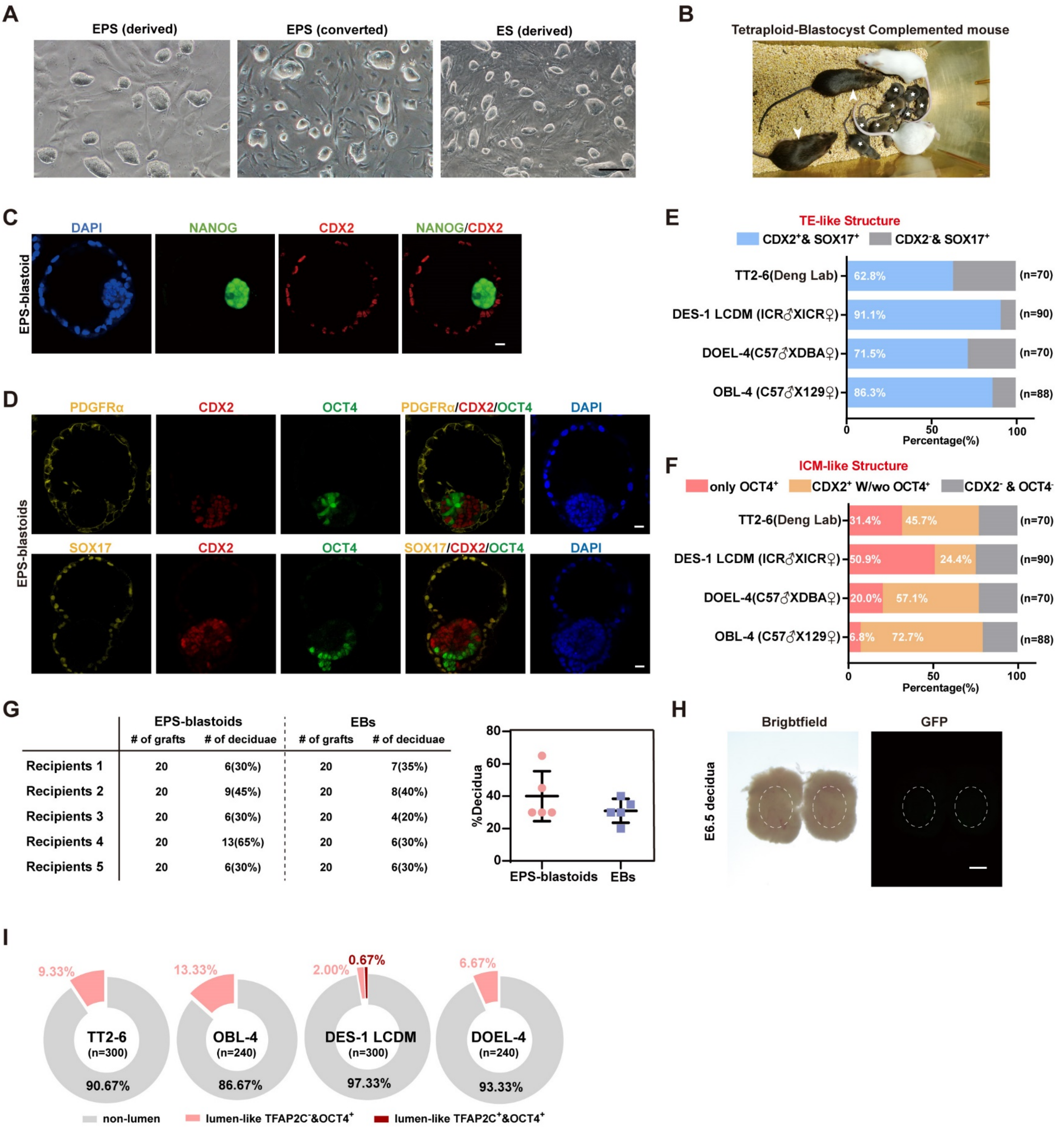

	EPS-blastoids		EBs	
	# of grafts	# of deciduae	# of grafts	# of deciduae
Recipients 1	20	6(30%)	20	7(35%)
Recipients 2	20	9(45%)	20	8(40%)
Recipients 3	20	6(30%)	20	4(20%)
Recipients 4	20	13(65%)	20	6(30%)
Recipients 5	20	6(30%)	20	6(30%)

Figure S1

Figure S1. Verification of the pluripotency of EPS cells and the development of EPS-blastoids after implantation, Related to Figure 1

(A) Representative images showing the clones morphology of EPS cells (derived from blastocyst or converted from ES cells) and ES cells derived from blastocyst. Scale bar, 100 μm.

(B) Brightfield image of EPS cells-derived mice (arrow-marked) via the tetraploid complementation method, and its offspring (asterisk-marked) produced by mated with ICR females.

(C and D) Immunofluorescence staining of EPS-blastoids. Staining for NANOG (EPI), OCT4 (EPI), CDX2 (TE), PDGFRα (PrE), SOX17 (PrE). Scale bars, 20 μm.

(E) The percentage of different TE-like structure categories of EPS-blastoids formed by different cell lines based on the expression of CDX2 and SOX17.

(F) The percentage of different ICM-like structure categories of EPS-blastoids formed by different cell lines based on the expression of OCT4 and CDX2. W/wo: with or without.

(G) The efficiency of induced decidua by EPS-blastoids and EBs (right). Data are represented as mean ± SD.

(H) Brightfield (left) and fluorescent (right) images of dissected E6.5 decidua induced by EPS-blastoid (GFP-marked). Scale bar, 100 μm.

(I) Pie charts showing the frequency of different IVC product categories of EPS-blastoids formed by different cell lines based on the expression of TFAP2C and OCT4.

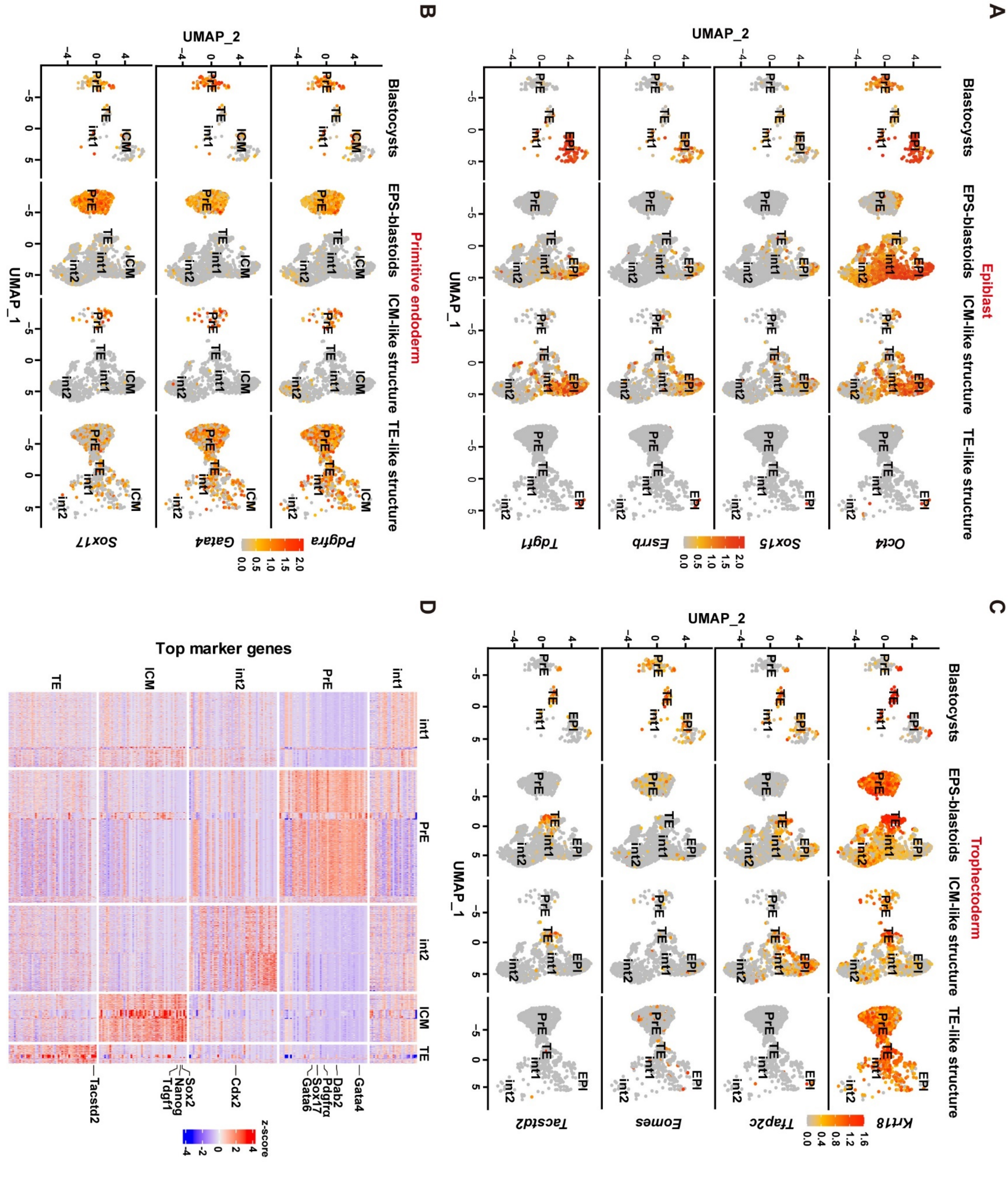

A
B
C
D
Epiblast
Primitive endoderm
Trophectoderm
Blastocysts
EPS-blastoids
ICM-like structure
TE-like structure
UMAP_1
UMAP_2
PrE
TE
ICM
EPI
int1
int2
Oct4
Sox15
Esrrb
Tdgf1
Pdgfra
Gata4
Sox17
Krt18
Tfap2c
Eomes
Tacstd2
Top marker genes
z-score
Gata4
Dab2
Pdgfrα
Sox17
Gata6
Cdx2
Sox2
Nanog
Tdgf1
Tacstd2

Figure S2

Figure S2. Expression of lineage-specific markers from scRNA-seq data, Related to Figure 2

(A-C) UMAP embedding of EPS-blastoids and blastocysts. Shown is the expression of three lineage markers for Epiblast (A), Primitive endoderm (B) and Trophectoderm (C), respectively. int-1: intermediate state 1, int-2: intermediate state 2.

(D) Heat map indicating the expression of lineage-specific genes in different clusters. For visualization purposes, the top 50 marker genes were shown, and ordered by significance.

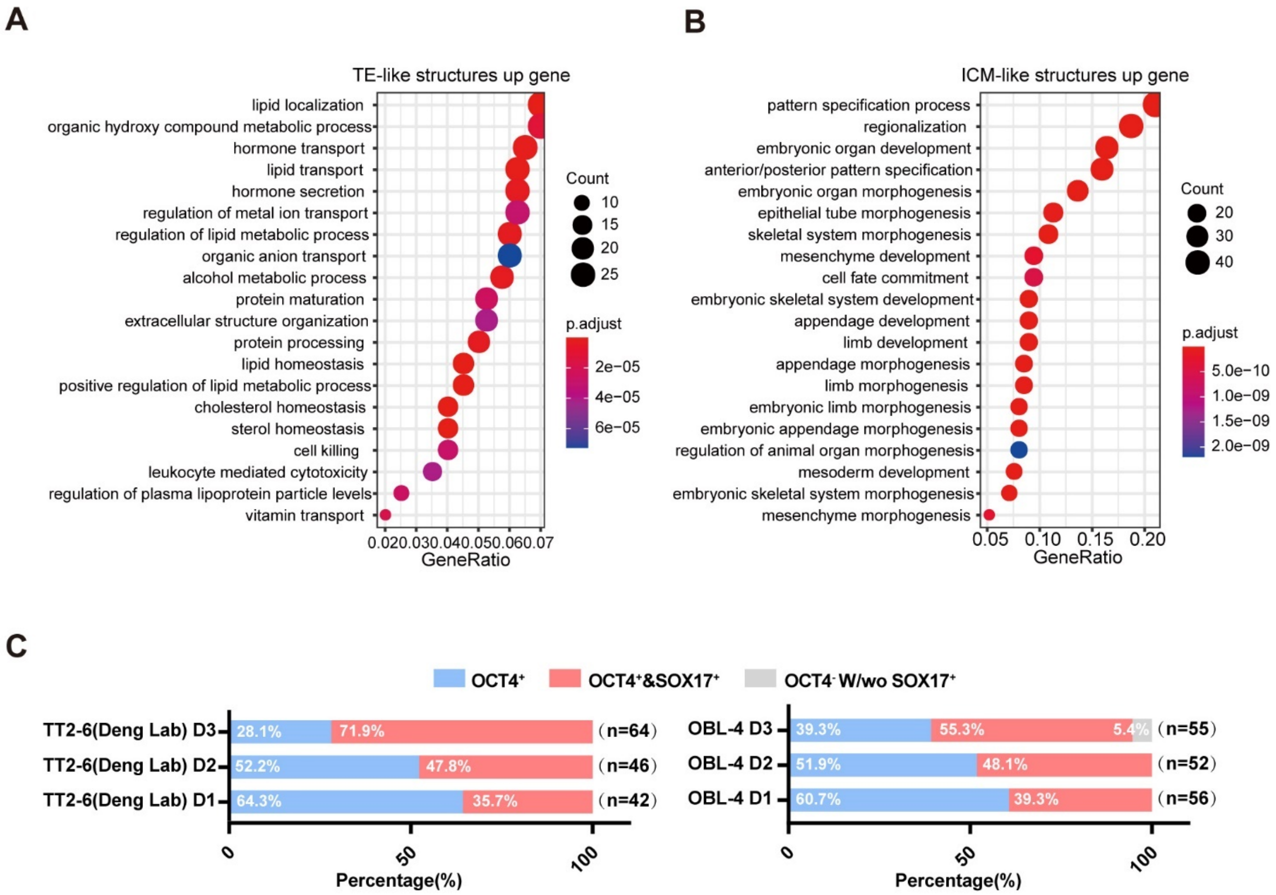
A
TE-like structures up gene
lipid localization
organic hydroxy compound metabolic process
hormone transport
lipid transport
hormone secretion
regulation of metal ion transport
regulation of lipid metabolic process
organic anion transport
alcohol metabolic process
protein maturation
extracellular structure organization
protein processing
lipid homeostasis
positive regulation of lipid metabolic process
cholesterol homeostasis
sterol homeostasis
cell killing
leukocyte mediated cytotoxicity
regulation of plasma lipoprotein particle levels
vitamin transport
0.020.030.040.050.060.07
GeneRatio
Count
10
15
20
25
p.adjust
2e-05
4e-05
6e-05
B
ICM-like structures up gene
pattern specification process
regionalization
embryonic organ development
anterior/posterior pattern specification
embryonic organ morphogenesis
epithelial tube morphogenesis
skeletal system morphogenesis
mesenchyme development
cell fate commitment
embryonic skeletal system development
appendage development
limb development
appendage morphogenesis
limb morphogenesis
embryonic limb morphogenesis
embryonic appendage morphogenesis
regulation of animal organ morphogenesis
mesoderm development
embryonic skeletal system morphogenesis
mesenchyme morphogenesis
0.05 0.10 0.15 0.20
GeneRatio
Count
20
30
40
p.adjust
5.0e-10
1.0e-09
1.5e-09
2.0e-09
C
OCT4⁺ OCT4⁺&SOX17⁺ OCT4⁻ W/wo SOX17⁺
TT2-6(Deng Lab) D3 28.1% 71.9% (n=64)
TT2-6(Deng Lab) D2 52.2% 47.8% (n=46)
TT2-6(Deng Lab) D1 64.3% 35.7% (n=42)
0 50 100
Percentage(%)
OBL-4 D3 39.3% 55.3% 5.4% (n=55)
OBL-4 D2 51.9% 48.1% (n=52)
OBL-4 D1 60.7% 39.3% (n=56)
0 50 100
Percentage(%)

Figure S3

Figure S3. Functional analysis of genes up-regulated in TE-like structure and ICM-like structure, and the cell lineage changes with the formation of blastoids. Related to Figure 3

(A and B) GO analysis of genes up-regulated in TE-like structure (A) and ICM-like structure (B).

(C) The percentage of different aggregation product categories at different points in time (day1, day2, and day3) based on the expression of OCT4 and SOX17.

A

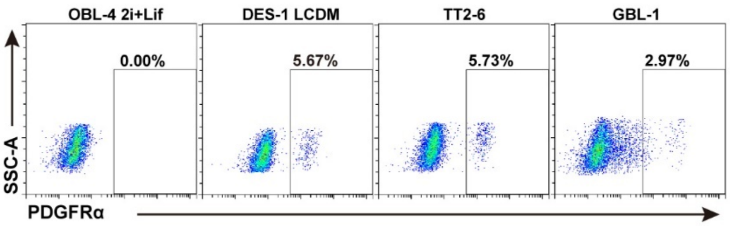

Figure S4

Figure S4. FACS analysis of the PrE-like cells in EPS cell lines, Related to Figure 4

(A) Representative FACS analysis of the percentages of PDGFRα$^+$ cells in ES cell line (OBL-4 2i+Lif) and EPS cell lines (DES-1 LCDM, TT2-6 and GBL-1).

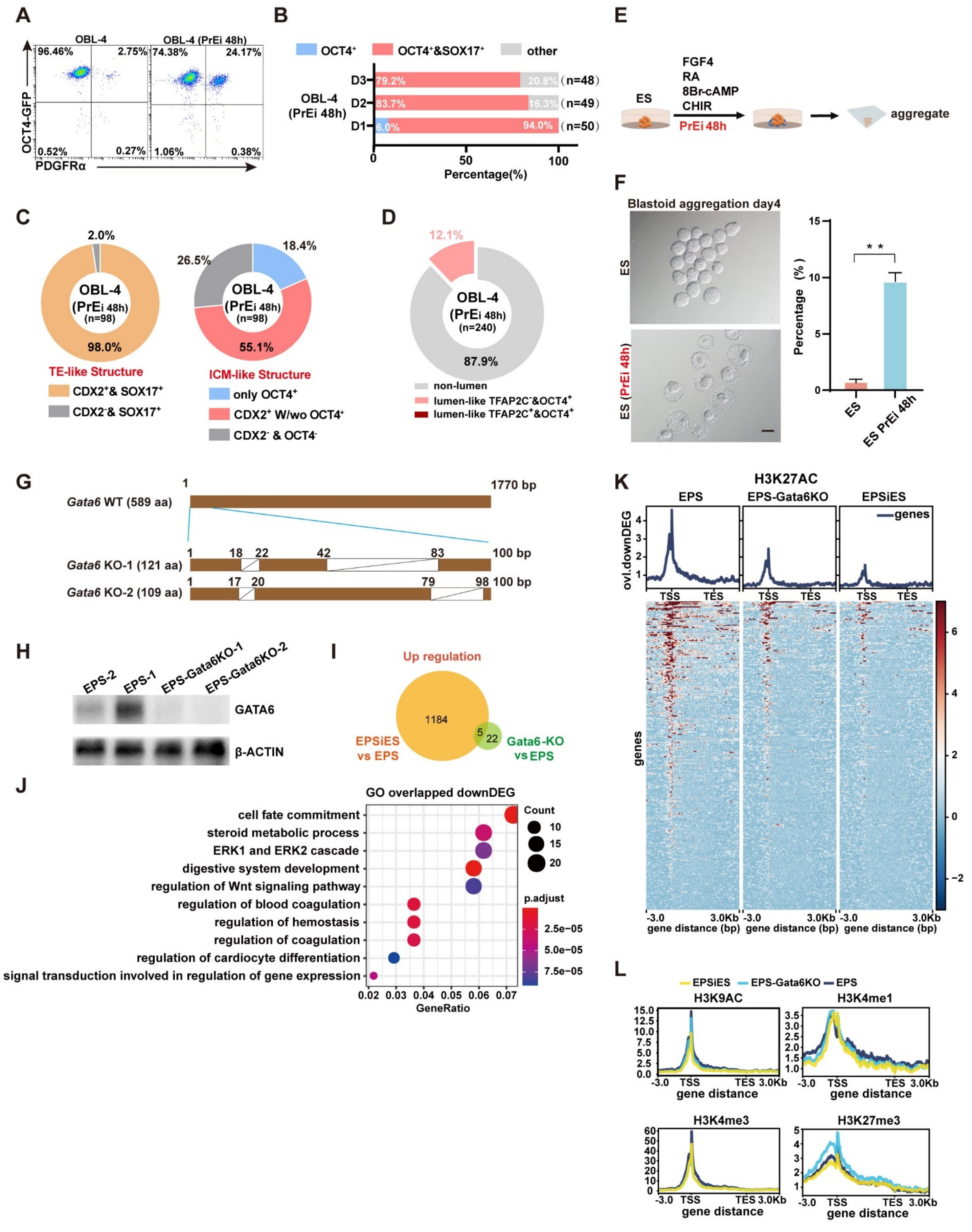
A
OBL-4
OBL-4 (PrEi 48h)
96.46%
2.75%
74.38%
24.17%
0.52%
0.27%
1.06%
0.38%
OCT4-GFP
PDGFRα
B
OCT4+
OCT4+&SOX17+
other
OBL-4 (PrEi 48h)
D3
D2
D1
79.2%
20.8%
(n=48)
83.7%
16.3%
(n=49)
6.0%
94.0%
(n=50)
Percentage(%)
E
ES
FGF4
RA
8Br-cAMP
CHIR
PrEi 48h
aggregate
C
OBL-4 (PrEi 48h) (n=98)
2.0%
98.0%
TE-like Structure
CDX2+& SOX17+
CDX2-& SOX17+
26.5%
18.4%
55.1%
ICM-like Structure
only OCT4+
CDX2+ W/wo OCT4+
CDX2- & OCT4-
D
12.1%
OBL-4 (PrEi 48h) (n=240)
87.9%
non-lumen
lumen-like TFAP2C-&OCT4+
lumen-like TFAP2C+&OCT4+
F
Blastoid aggregation day4
ES
ES (PrEi 48h)
Percentage (%)
ES
ES PrEi 48h
G
Gata6 WT (589 aa)
1770 bp
Gata6 KO-1 (121 aa)
Gata6 KO-2 (109 aa)
100 bp
H
EPS-2
EPS-1
EPS-Gata6KO-1
EPS-Gata6KO-2
GATA6
β-ACTIN
I
Up regulation
1184
5
22
EPSiES vs EPS
Gata6-KO vs EPS
J
GO overlapped downDEG
cell fate commitment
steroid metabolic process
ERK1 and ERK2 cascade
digestive system development
regulation of Wnt signaling pathway
regulation of blood coagulation
regulation of hemostasis
regulation of coagulation
regulation of cardiocyte differentiation
signal transduction involved in regulation of gene expression
Count
p.adjust
GeneRatio
K
H3K27AC
EPS
EPS-Gata6KO
EPSiES
genes
ovl.downDEG
TSS
TES
-3.0
3.0Kb
gene distance (bp)
L
EPSiES
EPS-Gata6KO
EPS
H3K9AC
H3K4me1
H3K4me3
H3K27me3
gene distance

Figure S5

Figure S5. Promotion of EPS-blastoid formation by PrE differentiation, Related to Figure 5

(A) Representative FACS analysis of the percentages of $OCT4^{+}PDGFR\alpha^{+}$ cells in OBL-4 and OBL-4 PrEi (PrE induction medium treat for 48 hours).

(B) The percentage of different aggregation product categories at different points in time (day1, day2, and day3) based on the expression of OCT4 and SOX17.This aggregation product formed by OBL-4 PrEi (PrE induction medium treat for 48 hours).

(C) Pie charts showing the frequency of different TE-like structure (left) and ICM-like structure (right) categories of EPS-blastoids formed by OBL-4 PrEi (PrE induction medium treat for 48 hours) based on the expression of CDX2, SOX17 and OCT4.

(D) Pie chart showing the frequency of different IVC product categories of blastoids fromed by OBL-4 PrEi (PrE induction medium treat for 48 hours) based on the expression of TFAP2C and OCT4.

(E) Schematic showed the process of blastoids aggregation of ES cells treated with PrE induction medium (N2B27 supplemented with FGF4, Retinoic acid, 8Br-cAMP and CHIR) for 48 hours.

(F) The representative morphology images of product aggregated by ES cells before and after PrE induction medium treatment (left), quantification of blastoids formation efficiency with ES cells under these two conditions (right). Data are represented as mean ± SD; ***P<0.001, unpaired student's t test. Scale bar, 100 μm.

(G) The genotypes of EPS-Gata6KO-1 and EPS-Gata6KO-2 cell line.

(H) Western blot analysis for the GATA6 protein levels in the EPS-2, EPS-1, EPS-Gata6KO-1 and EPS-Gata6KO-2 cells.

(I) Venn diagram for the number of shared up-regulated genes between the indicated groups.

(J) GO analysis of the shared down-regulated genes (303 genes, Shown in the figure 5N).

(K) H3K27ac signals at the TSS of shared down-regulation genes (303 genes, Shown in the figure 5N) in EPS, EPS-Gata6KO and EPSiES, represented as normalized RPKM

values.

(L) Average H3K9AC, H3K4me1, H3K4me3 and H3K27me3 signals at the TSS of shared down-regulation genes (303 genes, Shown in the figure 5N) in EPS, EPS-Gata6KO and EPSiES, represented as normalized RPKM values.

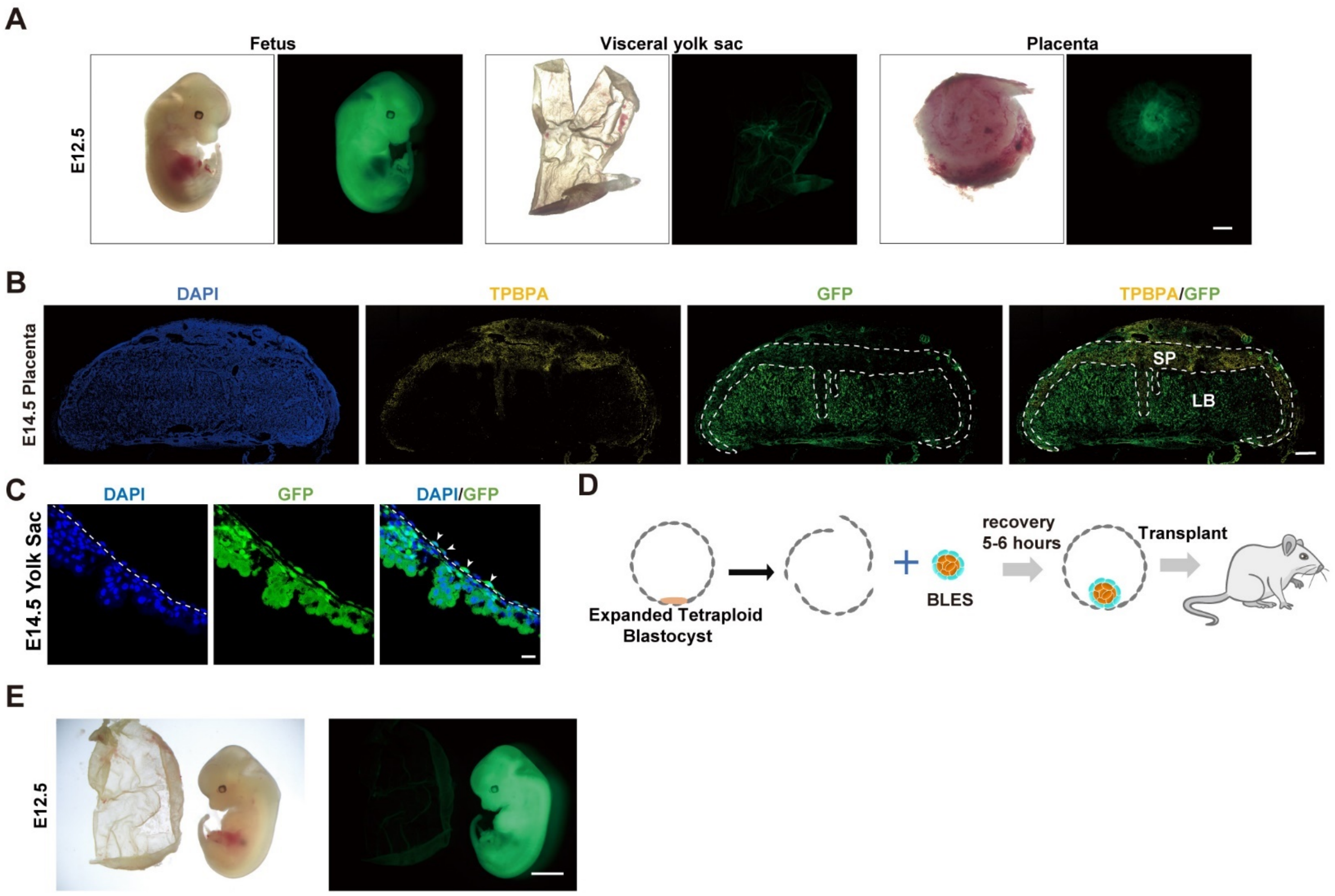
A
Fetus
Visceral yolk sac
Placenta
E12.5
B
DAPI
TPBPA
GFP
TPBPA/GFP
E14.5 Placenta
SP
LB
C
DAPI
GFP
DAPI/GFP
E14.5 Yolk Sac
D
Expanded Tetraploid
Blastocyst
BLES
recovery
5-6 hours
Transplant
E
E12.5

Figure S6

Figure S6. Verification of bidirectional pluripotency of BLES, Related to Figure 6

(A) Representative images of E12.5 chimeric embryos (containing a fetus, visceral yolk sac and placenta) produced by method in Figure 6E. Scale bar, 1000 μm.

(B) Immunofluorescence staining of placenta of E14.5 chimeric embryos produced by method in Figure 6E. Staining for DAPI (nucleus), GFP (doner cells) and TPBPA (spongiotrophoblast layer). LB: labyrinth layer, SP: spongiotrophoblast layer. Scale bar, 500 μm.

(C) Immunofluorescence staining of yolk sac of E14.5 chimeric embryos produced by method in Figure 6E. Staining for DAPI (nucleus) and GFP (doner cells). Scale bar, 20 μm.

(D) Schematic showed the procedure of chimeras. Firstly, TE of tetraploid embryos at E4.5 period were digested into sheets, then aggregated with BLES (produced with marked GFP EPS cells treated by PrE induction medium for 48 hours) of day1-2. After recovering for 5-6 hours, the chimeric embryos were transplanted into the uterus of a 2.5 dpc pseudo-pregnant mouse.

(E) Representative images of E12.5 chimeric embryo produced by above method (Figure S6D). Scale bar, 2000 μm.
